# Unsaturated Macrolactones from Renewable Feedstocks: Synthesis, Ring-Opening Polymerization and Application Prospects

**DOI:** 10.3390/ijms26115039

**Published:** 2025-05-23

**Authors:** Ilya Nifant’ev, Anna Afanaseva, Alexander Vinogradov, Pavel Ivchenko

**Affiliations:** 1 A.V. Topchiev Institute of Petrochemical Synthesis RAS, 29 Leninsky Pr., 119991 Moscow, Russia; aan.naa00@mail.ru (A.A.); amvvin@mail.ru (A.V.); 2 Chemistry Department, M.V. Lomonosov Moscow State University, 1 Leninskie Gory Str., Building 3, 119991 Moscow, Russia; 3 Faculty of Chemistry, National Research University Higher School of Economics, Myasnitskaya Str. 20, 101100 Moscow, Russia

**Keywords:** carbene complexes, grafted polymers, metathesis, metathesis polymerization, molybdenum catalysts, polyesters, ring-opening polymerization, ruthenium catalysts, unsaturated macrolactones

## Abstract

Unsaturated macrolactones (UMs) have long attracted researchers’ attention due to a combination of a reactive ester fragment and C=C bond in their structures. UMs of natural origin are comparatively few in number, and the task of developing synthetic approaches to new UMs is relevant. Recent advances in the synthesis of UMs cannot be dissociated from the progress in design of metathesis catalysts, since this catalytic approach is an atom-economy alternative to conventional organochemical methods. In the present review, we summarized and discussed the use of ring-closing metathesis, catalyzed by Ru and Group 6 metal complexes, in the synthesis of Ums and the advantages and shortcomings of the catalytic approach to UMs in comparison with organochemical methods. In a separate section, the use of UMs in the synthesis of unsaturated polyesters, the functionalization of these (co)polymers, and the prospects for practical use of the material obtained are also presented. It is essential that the actual approaches to UMs are often based on the use of renewable feedstocks, thereby meeting Green Chemistry principles.

## 1. Introduction

Unsaturated macrocyclic lactones (macrolactones, UMs) have long attracted the attention of researchers. As such, UMs are widely used as a components of perfume and cosmetic compositions [1]. At the same time, in recent years UMs are actively studied as (co)monomers in catalytic ring-opening transesterification polymerization (ROTEP) in order to obtain polyesters containing reactive C=C bonds in the main chain of (co)polymers [1,2]. Some UMs occur naturally in plants and animal organs [1]; however, further expansion of the range of similar compounds requires the development of new, atom-efficient and versatile synthetic approaches to these compounds.

**Scheme 1:**
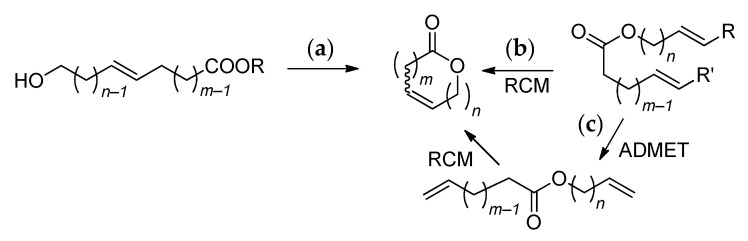
Common synthetic approaches to unsaturated macrolactones: (**a**) intramolecular (trans)esterification and (**b**) ring-closing metathesis (RCM) of alkenyl alkenoates, including RCM of acyclic diene metathesis products (**c**).

Common organochemical approaches to UMs primarily use a lactonization reaction, intramolecular (trans)esterification with the formation of unsaturated cyclic esters (Scheme 1a) [3,4,5]. The ring-closing metathesis (RCM) of alkenyl alkenoates (Scheme 1b) represents an obvious alternative to macrolactonization [6,7,8], and that is the direction that is being actively developed in recent years [7]. It is quite obvious that the metathesis of alkenyl alkenoates at high substrate concentrations mainly results in the formation of polymeric acyclic diene metathesis (ADMET) products (Scheme 1c), which in turn can be transformed into UMs by shifting the ring–chain equilibrium [9].

In our review, we describe and discuss synthetic approaches to UMs, focusing on the preparation of natural macrolactones and the RCM of alkenyl alkenoates based on renewable feedstocks. To complete the picture, actual data on the RCM of other alkenyl alkenoates are summarized to reveal key patterns and prospects for this catalytic process. Particular attention is paid to the results of recent studies aimed at the design of new Ru-, Mo- and W-based RCM catalysts and the development of efficient preparative-scale RCM reactions. The use of UMs in cosmetics and perfumery is beyond the scope of our review, and the last part of the manuscript is devoted to the use of UMs in the synthesis of unsaturated polyesters and the applications of the (co)polymers obtained.

## 2. Synthesis of Unsaturated Macrolactones

**Scheme 2:**
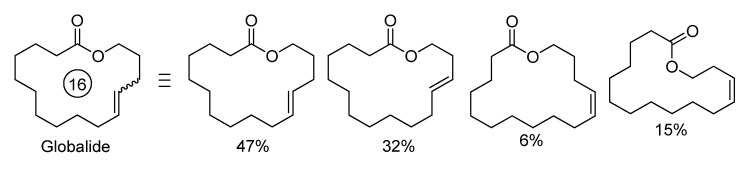
The real composition of commercial UM globalide (habanolide) [10].

The main problem for the further use of UMs, directly isolated from natural feedstocks, results from the variability in composition and structure of similar products. So, for example, commercial macrolactone globalide (formally corresponding to an oxacyclohexadec-12-en-2-one structure) is actually a mixture of positional and configurational isomers (Scheme 2) [10]. The use of other commercial names (e.g., habanolide) for the same product did not add clarity. First isolated in 1927 by M. Kerschbaum from ambrette (*Hibiscus abelmoschus*) seeds [11], ambrettolide (16-hexadec-7-enolide) was contained in the monoester fraction of ambrette seeds in an amount of 11 wt% (3.8‰ in dried seeds) along with 14-tetradec-5-enolide (0.5 wt%) and 18-octadec-9-enolide (7 wt%) [12], which makes them difficult to separate.

It is therefore apparent that the search for synthetic approaches to UMs has drawn researchers’ attention. An additional incentive for studies in this field is the possibility of obtaining new, previously unknown substances. To date, a number of conventional organochemical approaches to macrolactones have been developed [3,4,5]; however, in recent years more and more attention has been given to the catalytic ring-closing metathesis (RCM) of alkenyl alkenoates [7,8].

### 2.1. Organochemical Approaches to Unsaturated Macrolactones

A detailed discussion of organochemical approaches to UMs is not a subject for the present review. Below, we consider some particular examples of the preparation of Ums, with their advantages, disadvantages and limitations.

The methods and approaches of classical organic chemistry are not always able to provide the required structural homogeneity. For instance, cyclohexadeca-1,8-diene was converted via the intermediate formation of the corresponding unsaturated ketones and subsequent Baeyer–Villiger oxidation (8 stages) to a mixture of 17-membered unsaturated lactones (total yield 9.5%) [13]. Back in 1980, the WCl_6_/Me_4_Sn-catalyzed cross-metathesis of methyl undec-10-enoate and CH_2_=CH(CH_2_)_4_OAc, followed by hydrolysis and intramolecular esterification, was used in the synthesis of 15-membered unsaturated lactone; the yields of the reaction intermediates and final product were not reported [14]. Taking into account the statistical nature of cross-metathesis, this approach appears to be too costly.

**Scheme 3:**
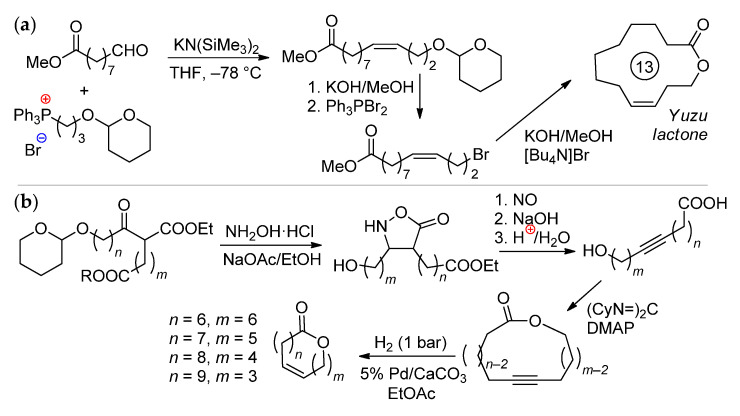
Organochemical approaches to unsaturated macrolactones. (**a**) Preparation of the Yuzu lactone [15]. (**b**) (*Z*)-selective synthesis of 10-membered lactones [16].

The synthesis of Yuzu lactone was based on the product of the ozonolysis of methyl oleate (MO); its interaction with phosphonium salt and KN(SiMe_3_)_2_ resulted in the formation of a pure (*Z*)-isomer of O-protected unsaturated ester (Scheme 3a). Its saponification, bromination and cyclization led to the formation of the target product (total yield 34%, *Z*:*E* ratio 80:1) [15]. A number of (*Z*)-unsaturated lactones were prepared from tetrahydropyranyl derivatives of ω-hydroxy keto esters via the intermediate formation of macrolactones with an internal C≡C bond (Scheme 3b) [16]. ω-Hydroxyalkynyl carboxylic acid intermediate (*n* = 5, *m* = 8) can be used in the synthesis of ambrettolide, first performed in 1983 [17]. An alternative approach to ambrettolide was based on the Wittig reaction between *^t^*BuMe_2_SiO(CH_2_)_8_CHO and [Ph_3_P(CH_2_)_6_COOMe]Br, followed by the deprotection and intramolecular esterification of ω-hydroxyalkenyl acid (1 mM in cyclohexane) using lipase B (*Cattleya aurantiaca*) acrylic resin as a catalyst; after 3 h at 40 °C, the yield was 40% [18]. In a recent study of Guerrero-Morales and Collins, a preference for the biocatalytic route to macrolactones was demonstrated [19]. Although high yields in the macrolactonization of ω-hydroxyacids can be provided by the use of C_6_F_5_COCl via the intermediate formation of mixed anhydrides, high dilutions (~1.5 mM) are still needed [20].

**Scheme 4:**
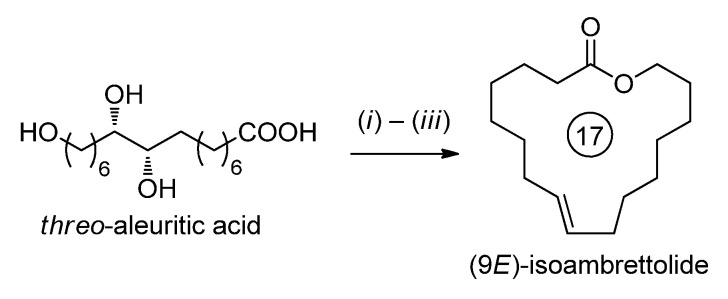
Synthesis of (9*E*)-isoambrettolide. Reagents and conditions: (*i*) 2-methyl-6-nitrobenzoic anhydride, *N*,*N*-dimethylaminopytidine *N*-oxide/Et_3_N in THF/CH_2_Cl_2_, 83%; (*ii*) 1,10-thiocarbonyldiimidazole/*N*,*N*-dimethylaminopytidine, toluene, 110 °C, 91%; (*iii*) P(OMe)_3_, 140 °C, 87% [21].

Based on *threo*-aleuritic acid, a major ingredient of the *Laccifer lacca* natural shellac, (9*E*)-isoambrettolide was synthesized (total yield 72%, Scheme 4) [21].

**Scheme 5:**
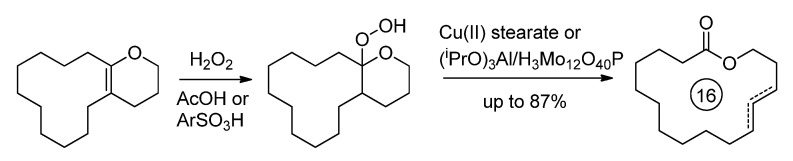
Synthesis of 16-membered UMs from dodecahydro-2*H*-cyclododeca[*b*]pyran [23,24].

Among other organochemical approaches to different UMs, the synthesis of 16-membered macrolactone impresses with its simplicity and the availability of the starting compound, dodecahydro-2*H*-cyclododeca[*b*]pyran [22]. The corresponding hydroperoxide was converted into corresponding UMs with high yields (Scheme 5) [23,24].

Except for the last approach, which has severe limitations on the substrate and corresponding product, organochemical methods of the synthesis of UMs are complex, have a low atom efficiency and are unable to provide preparations of the wide spectrum of macrolactones. At first glance, the ring-closing metathesis of alkenyl alkenoates appears to be more convenient and versatile, but certain difficulties arise when using RCM for macrolactonization.

### 2.2. The Basic Patterns of Ring-Closing Metathesis

**Scheme 6:**
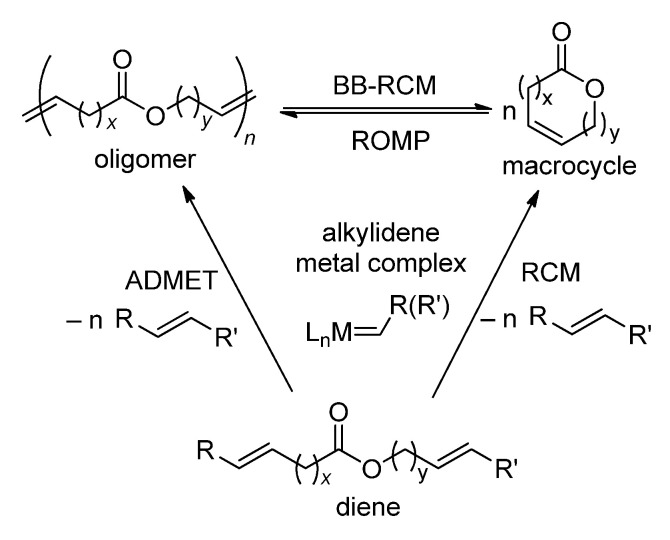
Transformations of alkenyl alkenoates in the presence of metathesis catalysts.

Scheme 6 reflects the main processes involving alkenyl alkenoates in the presence of metathesis catalysts, metal carbene complexes. The substrates can react with a formation of UMs (RCM, intramolecular process) or oligomers/polymers, the products of acyclic diene metathesis polymerization (ADMET, intermolecular process). To minimize the formation of oligomers, the reaction should be conducted according to the Ruggli–Ziegler high dilution principle, with a substrate concentration ~1 mmol∙L^−1^ (mM). The nature of the catalyst is essential for achieving the high productivity and selectivity of the process. The nature of the substrate is also important; however, alkenyl esters of both oleic acid and ω-unsaturated acids (dec-9-enoic, undec-10-enoic, etc.) are very attractive for using in RCM due to the high synthetic availability of these substrates. In particular, methyl oleate (MO) is the main component of fatty acid methyl esters (FAMEs) obtained by the methanolysis of high-oleic plant oils [8], undec-10-enoic acid is produced industrially from castor oil [25], and methyl dec-9-enoate can be obtained by the ethenolysis of MO [26,27,28]. It is these alkenyl esters that are of the greatest interest as a part of this review.

In contrast to the industrially implemented cross-metathesis of ethylene and α-olefins, based on the use of moderately active catalysts under harsh conditions [29], the RCM of alkenyl alkenoates requires the use of active catalysts, carbene complexes of Ru and Mo. It should be noted though that Ru- and Mo-catalyzed RCM at elevated temperatures, with the distillation of the reaction products, was developed in recent years (see Section 2.4).

**Scheme 7:**
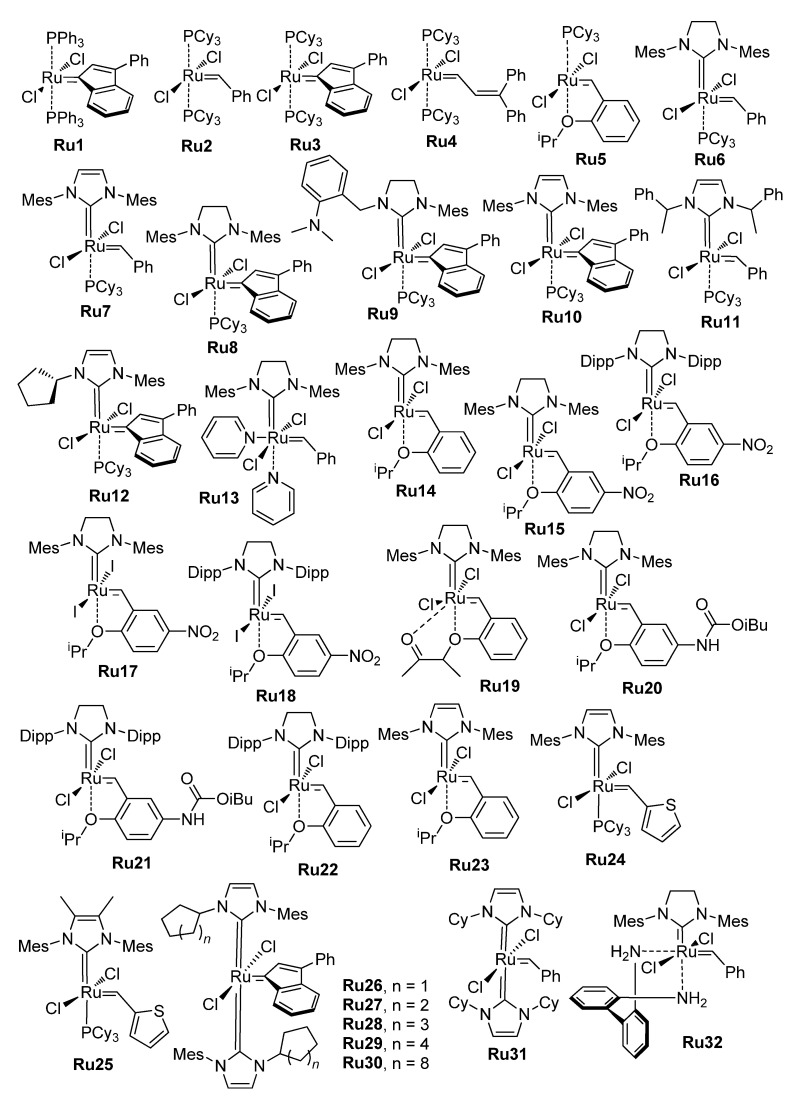

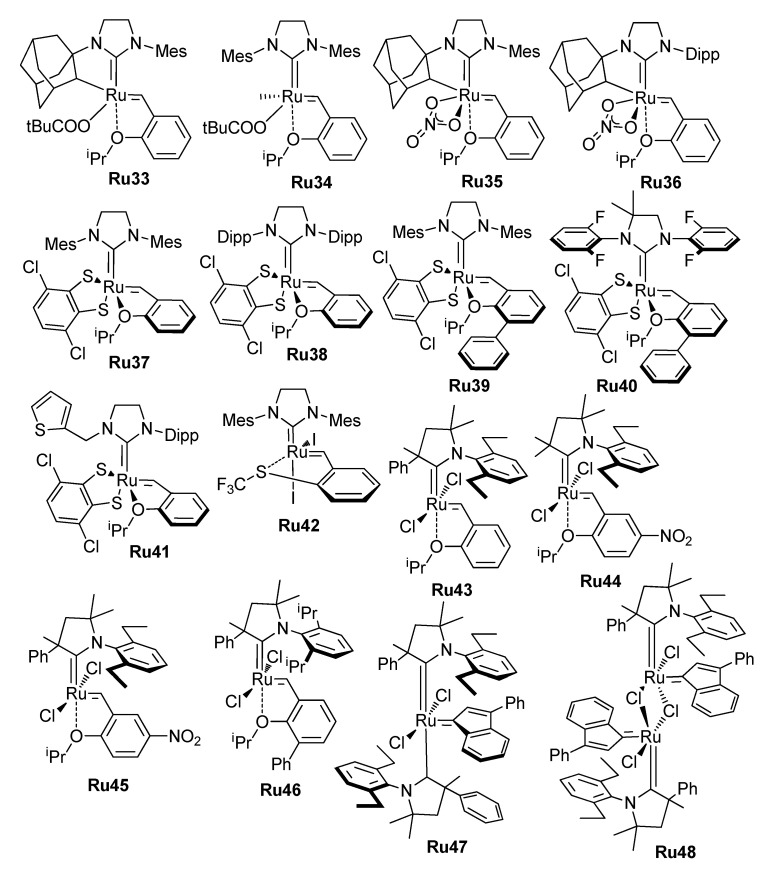
Ru catalysts studied in RCM of alkenyl alkenoates.

The high catalytic activity of Ru and Mo carbene complexes also has a reverse side, which is that the active catalysts have increased sensitivity to substrate impurities, moisture and air, and the progress in RCM was inextricably linked with the common progress in the design of chemically stable metathesis catalysts. The structural formulas of Ru complexes, studied in the RCM of alkenyl alkenoates and related substrates, are presented in Scheme 7. The first-generation Grubbs catalysts **Ru1**–**Ru4** (Grubbs I, G-I) turned out to be relatively unstable; the Grubbs II (G-II) catalysts **Ru6**–**Ru8** and **Ru10**, as well as the Hoveyda–Grubbs II (HG-II) catalyst **Ru14**, showed better results (see Section 2.3.1). Even more efficient RCM catalysts have been synthesized and studied in recent years (see Section 2.3 and Section 2.4). The Group 6 metal (Mo, W)-catalyzed RCM of alkenyl alkenoates was the subject of a few studies (see Section 2.5).

**Scheme 8:**
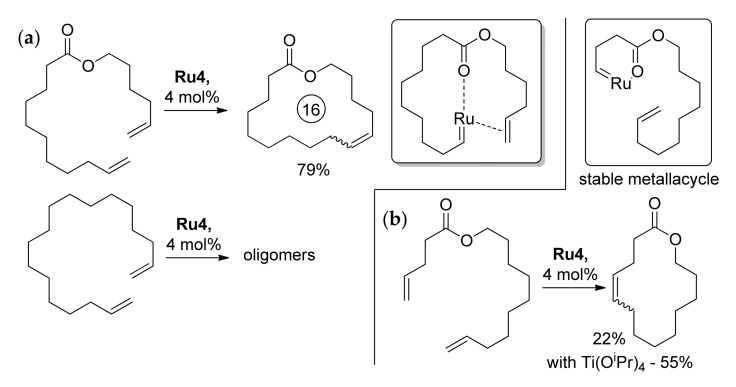
(**a**) Successful RCM of hex-5-enyl undec-10-enoate due to the presence of the ester group and intermediate >C=O^…^Ru coordination [30]. (**b**) Formation of stable chelate that hinders RCM and the positive effect of the addition of Ti(O^i^Pr)_4_ [32].

The presence of the ester group in the substrate molecule could help to promote or hinder RCM. For example, in the presence of **Ru2** (4 mol%), hex-5-enyl undec-10-enoate formed 16-membered lactone with a 79% yield, whereas octadeca-1,17-diene formed oligomers (Scheme 8a) [30]. This observation can be explained by the additional coordination of >C=O at the Ru center, which creates a ‘template’ for cyclization [31]. At the same time, the presence of a relatively short (CH_2_)_n_ fragment between the ester group and double bond may result in catalyst inhibition due to the formation of stable chelates (Scheme 8b) [31,32,33]. A partial solution to the problem was the addition of Ti(O^i^Pr)_4_ that degrades chelate by the formation of the more stable >C=O^…^Ti bond [32]. In this way, the lengths of hydrocarbon spacers between ester group and C=C bonds affect the yield and selectivity of the RCM, and the synthetic availability of the UMs depends largely on the ring size (see Section 2.3.3).

Another important aspect of RCM is regio- and stereoselectivity [34]. The migration of the C=C bond during the reaction is caused by decomposition of the catalyst, with the formation of low-valent species capable of activating C–H bonds. The stereoselectivity ((*Z*)-/(*E*)- ratio) depends on the relative stability of the diastereomers; for lengthy Ums, (*Z*)-isomers are thermodynamically less stable than the corresponding (*E*)-isomers [34]. The nature of the catalyst can also affect the stereoselectivity, and studies in this area were actively carried out in 2000s [35] and are continuing at present (see Section 2.4).

**Scheme 9:**
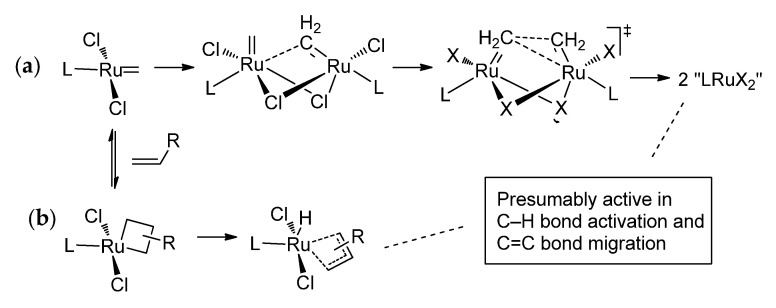
Mechanisms of decomposition of Ru metathesis catalysts, (**a**) via formation of L_n_Ru=CH_2_ species and (**b**) via β-hydride elimination [36,39].

In addition to spacing between the ester fragment and C=C bonds, the type of the alkenyl fragments in the substrate is important: when alkenyl alkenoate represents α,ω-diene, ethylene is a product of the RCM process. The formation of ethylene, with its subsequent elimination from the reaction mixture, would favor the RCM process. On the other hand, the intermediate formation of L_n_Ru=CH_2_ species can promote decomposition of Ru-based catalysts [36]. Generally, decomposition of Ru-based catalysts [37] can occur in two pathways, via bimolecular decomposition involving L_n_Ru=CH_2_ species and bimolecular coupling (Scheme 9a) or via β-hydride elimination (Scheme 9b), and the rate of decomposition directly depends on the ligand environment of the Ru center. Ru hydride complexes can also be formed during decomposition of the catalysts; similar species are also considered as C–H activation and C=C bond isomerization catalysts [38]. Combined experimental and theoretical studies of different Ru complexes revealed the lower stability of cyclic alkyl amino carbene (CAAC) complexes (for example, **Ru43**) in comparison with *N*-heterocyclic carbene (NHC) derivatives (HG-II complex **Ru14** and its analogs) in bimolecular decomposition. On the other side, CAAC-Ru complexes turned out to be more resistant to β-H elimination in comparison with NHC-Ru catalysts [39]. The influence of the nature of alkenyl fragments in substrate (terminal or not) on the yield of the UMs and reaction selectivity is discussed in Section 2.3.2.

To prevent isomerization, the use of different additives, e.g., chlorinated solvents [40], Brønsted acids [41] and substituted benzoquinones (for example, 2,3,5,6-tetrafluorocyclohexa-2,5-diene-1,4-dione, TFQ) [42,43], was proposed.

Numerous studies, aimed at the development of highly active and/or highly selective metathesis catalysts bearing in mind the mechanistic features of the process, have been conducted. A number of key patterns have been identified for simpler substrates, e.g., olefins. In particular, even in 2009 Schrock, Hoveyda and coll. revealed the impact of the bulky phenolate fragment in Mo carbene complexes on (*Z*)-selectivity in the homocoupling of α-olefins, attributed to the preference of the formation of less sterically hindered metallacyclic intermediates with *syn*-configuration than that form (*Z*)-alkene [44]. The appplicability of this approach to the synthesis of (*Z*)-UMs using Mo and W catalysts was demonstrated in later studies [45,46]. M=CH_2_ species promote secondary (*Z*)-alkene isomerization, and the elimination of ethylene is crucial for the high (*Z*)-stereoselectivity of RCM [46].

For Ru-based catalysts, the formation of constrained geometry centers with an unequal ligand environment provided a higher (*Z*)-selectivity. Examples of (*Z*)-selective Ru catalysts include **Ru35** and **Ru36** [47,48]; the further studies of (*Z*)-selective catalytic systems are discussed in Section 2.3.4.

**Figure 1:**
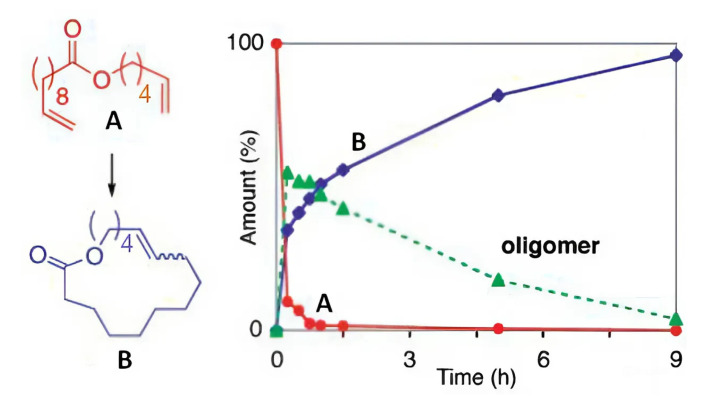
Metathesis of CH_2_=CH(CH_2_)_8_COO(CH_2_)_4_CH=CH_2_ (5 mol% **Ru7**): substrate conversion (A, red) and the content of oligomers (green) and RCM product (B, blue) in the reaction mixture over time. Adopted with permission from [49]. Copyright (2007) American Chemical Society.

It should be noted that, according to reference [49], the formation of UMs may be preceded by the formation of oligomeric ADMET products that will subsequently form macrocycles via backbiting RCM. As can be seen in Figure 1, in the presence of **Ru7** (5 mol%), a reduction in the concentration of oligomer is accompanied by a proportional increase in the concentration of 16-membered unsaturated lactone. This transformation takes place in time, making it important to increase the stability of the catalysts.

### 2.3. Ru-Catalyzed Ring-Closing Metathesis of Alkenyl Alkenoates at High Dilution Ratios

#### 2.3.1. Early Studies of Ru-Catalyzed RCM

During the late 1990s, some Ru-based catalysts were investigated. In 1996, Fürstner and Langemann synthesized isomeric 16-membered lactones from CH_2_=CH(CH_2_)_8_COO(CH_2_)_4_CH=CH_2_ and CH_2_=CH(CH_2_)_4_COO(CH_2_)_8_CH=CH_2_, in the presence of **Ru4** (4 mol%), with yields of 79 and 62%, respectively [50]. The same authors a year later showed that corresponding α,ω-dienes under similar conditions formed oligomers [30]. The cyclization of CH_2_=CH(CH_2_)_2_COO(CH_2_)_8_CH=CH_2_ (5 mol% of **Ru4**) proceeded with a 22% yield that was increased to 55% when Ti(O^i^Pr)_4_ (5 mol%) was added (Scheme 8b) [32].

The RCM of CH_2_=CH(CH_2_)_n_COO(CH_2_)_9_CH=CH_2_ (*n* = 7, 8), catalyzed by **Ru4** (1 mol%, C_6_H_6_, 60 °C), resulted in the formation of 20-membered (*Z*/*E* 57:43) and 21-membered (*Z*/*E* 60:40) macrolactones; the yields were 83 and 82%, respectively. ω-Alkenyl oleates gave 19- and 20-membered (*Z*/*E* 71:29) lactones with 65 and 63% yields. The concentrations of substrates were 5–7 mM [51]. Of undoubted interest are the results of comparative studies of the RCM of CH_2_=CH(CH_2_)_n_COO(CH_2_)_10−n_CH=CH_2_ (2 mM in CH_2_Cl_2_, 2 mol% **Ru4**), with the formation of 14-membered lactones [52]. As indicated in Table 1, maximum yields were achieved when using alkenyl alkenoate with C=C fragments equidistant from the ester group. Calculations of the relative strain of diastereomers using the MicroModel 4.5 program [53] showed that the (*Z*)/(*E*) ratio in most cases is determined by the relative stability of isomers. It should also be noted that allyl ester turned out to be inert in RCM.

The RCM of CH_2_=CH(CH_2_)_8_COO(CH_2_)_n_CH=CH_2_ (*n* = 4, 9) revealed the difference between **Ru11** and **Ru31**: the former was more efficient in the synthesis of 21-membered lactone, whereas the latter catalyzed cyclization to 16-membered lactone with a higher yield [54]. Double-bond isomerization with the formation of 20-membered lactone was detected back in 2000 in the example of CH_2_=CH(CH_2_)_8_COO(CH_2_)_9_CH=CH_2_ and **Ru7 [40]**. A study of the influence of the type of alkenyl fragment (terminal or internal) in CH_2_=CH(CH_2_)_8_COO(CH_2_)_2_CH=CHR (3 mM in CH_2_Cl_2_) on conversion and the (*Z*)/(*E*) ratio was also carried out in 2000 with the use of **Ru2** and **Ru6** (0.5–5 mol%); near quantitative yields were reported for **Ru6** without kinetic selectivity control [55]. At early stages of the studies, the use of supercritical CO_2_ was proposed for the RCM of CH_2_=CH(CH_2_)_8_COO(CH_2_)_4_CH=CH_2_ (2.7 mM); in the presence of **Ru4** (1 mol%) an 88% yield was achieved after 72 h at 40 °C [56].

In the 2000s, there was a decline in researchers’ interest in the RCM of alkenyl alkenoates; the only article [57] was devoted to the synthesis of alkenyl alkenoates CH_2_=CH(CH_2_)_n_COO(CH_2_)_n+1_CH=CH_2_ (*n* = 3–16) from CH_2_=CH(CH_2_)_n_CHO by the Tishchenko reaction (^i^Bu_2_AlH), followed by G-I (10 mol%)-catalyzed RCM. An increasing interest in RCM was observed in the 2010s and in recent years, with the development of new types of metathesis catalysts (see below).

#### 2.3.2. Different Ru Catalysts in RCM of Hex-5-en-1-yl Undec-10-enoate

Since the yield and selectivity of RCM depend not only on catalysts but also on the type of substrate (the length of (CH_2_)_n_ spacers between the ester group and C=C fragment, terminal or internal C=C bond), comparison of the catalysts makes sense when the same substrate is used in different experiments. Due to the high availability of undec-10-enoic acid, a large number of works were devoted to the study of the RCM of CH_2_=CH(CH_2_)_8_COO(CH_2_)_4_CH=CH_2_ [33,41,49,50,54,58,59,60,61,62,63,64,65,66]. The results of these studies are summarized in Table 2.

As can be seen in Table 2, G-I catalysts (**Ru2**, **Ru3**) were virtually inactive in the RCM of CH_2_=CH(CH_2_)_8_COO(CH_2_)_4_CH=CH_2_ [41], whereas the close analog **Ru4** provided relatively high yields of the UM but for a long reaction time [50]. G-II and HG catalysts containing saturated (e.g., **Ru6**, **Ru8**, **Ru14**–**Ru18**) and unsaturated (**Ru7**, **Ru23**–**Ru25**) NHC fragments turned out to be more active. The HG-II catalyst **Ru15**, containing an acceptor–NO_2_ group in the benzene ring, showed a higher productivity in comparison with **Ru14**; an 87% yield of the UM was achieved when using 0.05 mol% of the catalyst [62]. It was also reported that the use of R_2_SO as an additional ligand in G-II type catalysts, instead of PCy_3_, increases RCM productivity and selectivity [69]. The catalytic activity and thermal stability of the NHC-type catalysts were improved by the coordination of bidentate (N,N)-ligand at the Ru center (**Ru32** [33,62]). Among NHC Ru complexes, **Ru24** and **Ru25** have demonstrated the highest activities when ethylene was removed from the reaction mixture [61].

The first attempts to optimize the process parameters (reactor configuration) for the **Ru6**-catalyzed RCM of CH_2_=CH(CH_2_)_8_COO(CH_2_)_4_CH=CH_2_ were made in 2010 by Fogg and coll. [70]. The use of a continuous stirred-tank reactor with an efficient elimination of ethylene afforded an order-of-magnitude increase in TON and reduced the catalyst loadings to 0.2 mol%, thus providing near quantitative levels of yield and selectivity. The use of a continuous-flow reactor allowed the substrate concentration to be increased to 20 mM (C_2_H_4_Cl_2_); after 3 h at 70 °C in the presence of **Ru35** (7.5 mol%) the yield of UM was 70% ((*Z*)/(*E*) ratio 86:14) [71].

In recent years, CAAC Ru complexes have attracted researchers’ attention, demonstrating high activities in the cross-metathesis of oleates [26,27,28]. In the RCM of CH_2_=CH(CH_2_)_8_COO(CH_2_)_4_CH=CH_2_ the complexes **Ru43**–**Ru45** showed high activities; a 98% yield of UM was obtained when using 45 ppm of **Ru45**; a record TON of 62,000 was achieved at a 10 ppm loading of **Ru45 [59]**. However, the bis-CAAC complex **Ru47** was inferior to **Ru45** in catalytic activity [65]. The preference of CAAC complexes in RCM was confirmed by a mechanistic point of view in a recent study of Fogg and coll. [58]. Note that in 2019, the scientific group of Fogg reported that the dimeric CAAC complex **Ru48** is highly active in RCM (a 56% conversion after 2 h at 40 °C at a 20 ppm loading of the catalyst) [64]. Also note that the replacement of Cl by I in **Ru44** and other CAAC Ru complexes resulted in an increase in the yield of UM [68].

In most RCM experiments, the (*Z*)/(*E*) ratio was 1:2 to 1:4, reflecting the relative thermodynamic stability of diastereomers of 16-membered unsaturated lactone. However, the sterically hindered complexes **Ru35** and **Ru37** containing acceptor bidentate ligands (NO_3_^−^, 3,6-dichlorobenzene-1,2-ditiolate) showed a high (*Z*)-selectivity [62] (the stereoselectivity of RCM is discussed in more detail in Section 2.3.4)

As was mentioned in Section 2.2, the selectivity of RCM (the formation of the cycle with a given number of atoms) may be quite different from 100%: C=C bond migration during the RCM process may result in the formation of UMs with a smaller ring size. For example, the selectivities of the RCM of CH_2_=CH(CH_2_)_8_COO(CH_2_)_4_CH=CH_2_ (10 mM in EtOAc, 1 mol% of the catalyst) were 95% (**Ru6**), 98% (**Ru8**), 93% (**Ru14**), 77% (**Ru15**), 67% (**Ru19**), 96% (**Ru21**) and >99% (**Ru12**) [41]; isomerization was suppressed by the addition of 2–20 mol% HCl.

#### 2.3.3. Ru-Catalyzed RCM of Other Alkenyl Alkenoates

**Figure 2:**
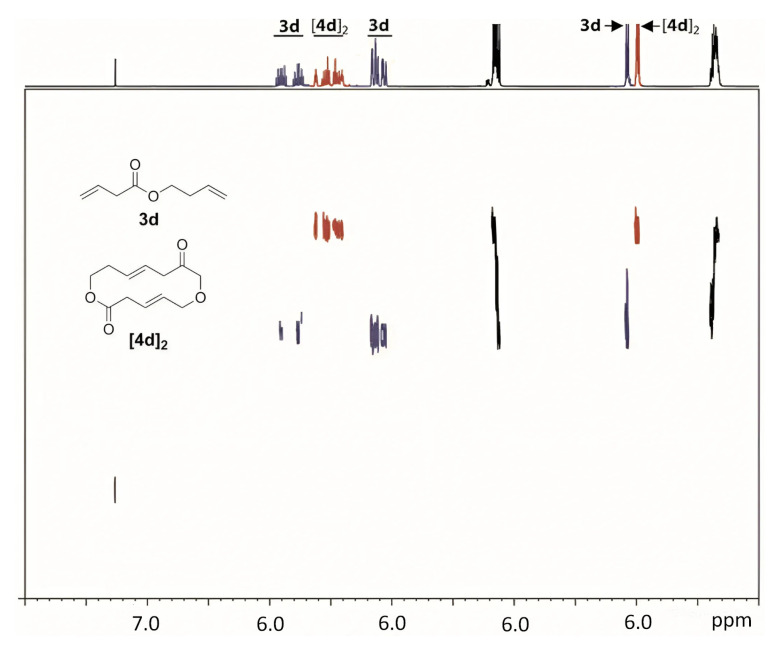
^1^H DOSY NMR spectrum of a 1:1 mixture of but-3-enyl but-3-enoate (**3d**) and its cyclic dimer, (4*E*,11*E*)-1,7-dioxacyclotetradeca-4,11-diene-2,9-dione (**[4d]_2_**) (500 MHz, CDCl_3_). The signals of **3d** and **[4d]_2_** are shown in blue and in red, respectively. Reprinted with permission from [73]. Copyright (2010) Elsevier B. V.

To date, a wide range of alkenyl alkenoates have been studied in Ru-catalyzed RCM. The diversity of the types of substrates and reaction products required the development of reliable methods for the analysis of reaction mixtures. The most common methods of analysis have been and remain GC (e.g., [41,70]) and GC/MS (e.g., [31,72]); ^1^H NMR diffusion-ordered spectroscopy (DOSY) can also be applied for the detection of the characteristic signals of RCM products and oligomers (Figure 2); these data can be used for the fast monitoring of the reaction mixtures [73].

The results of the studies of different catalysts and substrates are summarized in Table 3. The data presented do not include the results of the studies with the use of clearly unacceptable catalyst’s loadings and conditions (e.g., 20 mol% in [74], 10 mol% and 5 days of the reaction in [75]), without correctly specifying the catalysts [76] and suspect reports [77] (see the more detailed comment at the end of this section). As can be seen in Table 3, the yields and configurations of UMs depend on the lengths of alkenyl fragments and positions of C=C bonds and their configurations.

In general, the yields of 10–13-membered UMs are lower; the optimum ring size is 15–20. Note that six-membered unsaturated UM can be obtained via RCM, in contrast to seven-membered [49]. Undoubtedly of interest is the study of the RCM of dodec-11-en-1-yl acrylate (1 mM in C_6_H_6_): **Ru14** and **Ru35** (10 mol%) catalyzed the formation of 14-membered lactone with 87 and 5% yields, respectively [78]. The common patterns of the formation of UMs match those of the cyclization of bifunctional substrates [87], manifested in lower yields of 7–13-membered cyclic products. Back in 2007 [49], Fogg formulated recommendations for the optimal concentrations of α,ω-dienes in RCM: 100 mM for 5–6-membered rings, 5 mM for 7-membered, 0.5 mM for 8–13-membered, and 5 mM for 14+ macrocycles. With regard to unsaturated lactones, these recommendations apply only to 6-membered and 10+ macrocycles. The yields of UMs in some cases are relatively low, but in some cases RCM seems to be better than separation from the natural sources: for example, the content of 13-membered unsaturated lactone, derivative of dec-9-enoic acid, in *Medicago rugosa* amounted to ~8 mg∙kg^−1^ [88].

**Scheme 10:**
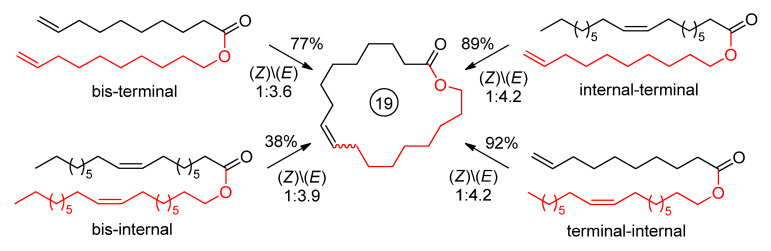
Influence of the substituents at the C=C bond on the yield of 19-membered UM [42].

The type of the alkenyl alkenoate (terminal or internal C=C fragment) can also affect the product yield and selectivity (the configuration of the C=C bond is also important, see Section 2.3.4). During a comparative study of four substrates, derivatives of oleic acid in **Ru15**-catalyzed RCM (Scheme 10), Grela and coll. showed the preference of the use of alkenyl alkenoates containing one terminal and one internal C=C bond [42]. In the synthesis of 16-membered UM, the type of C=C bond had little effect on the yield of the product [42].

**Figure 3:**
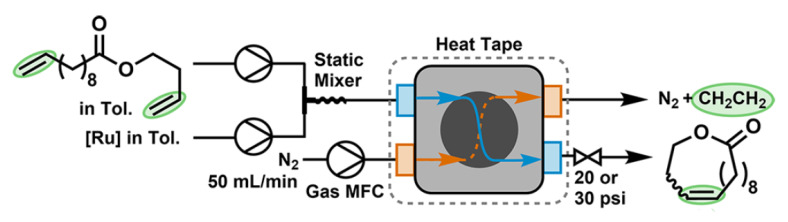
Continuous RCM of CH_2_=CH(CH_2_)_8_COO(CH_2_)_2_CH=CH_2_ using the membrane sheet-in-frame reactor. Reprinted with permission from [89]. Copyright (2020) American Chemical Society.

In 2020, Jamison, Bio and coll. proposed the use of a membrane sheet-in-frame pervaporation module reactor (Figure 3) for the RCM of CH_2_=CH(CH_2_)_8_COO(CH_2_)_2_CH=CH_2_; under optimized conditions (5 mM in toluene, 1.5 mol% **Ru17**, 10.5 min at 100 °C), the yield of UM was 95.4% [89].

At the conclusion of this section, the results of the catalytic experiments on the RCM of different alkenyl alkenoates, including allyl esters (50 mM in CH_2_Cl_2_), catalyzed by **Ru6** [77] should be mentioned. The reported isolated yields of unsaturated 11–23-membered lactones amounted to 72–85% which is contrary to the results of early and recent studies, and these data were not included in Table 3.

#### 2.3.4. Stereoselectivity of Ru-Catalyzed Ring-Closing Metathesis

**Scheme 11:**
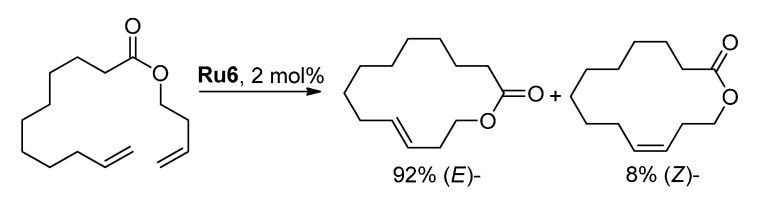
Stereoselective cyclization of but-3-enyl undec-10-enoate [55].

Besides the macrolactone yield and selectivity of RCM (the absence of C=C bond migration), the (*E*)/(*Z*) ratio is also important. In 2000, Lee and Grubbs showed that the formation of 14-membered lactone occurs with a high (*E*)-selectivity due to the higher thermodynamic stability of the (*E*)-isomer [55] (Scheme 11).

In the case of lactones with larger rings, the relative stability of (*Z*)-isomers increases, setting the stage for the development of stereoselective metathesis catalysts. The (*Z*)-selectivity of NHC Ru catalysts in the RCM of model substrates was increased by the coordination of the –OCMe_3_ ligand (**Ru33**, **Ru34**) [90,91]. Further studies [47,92] revealed cyclometallated catalysts with high (*Z*)-selectivity (**Ru35**, **Ru36**); the complex **Ru35** showed a high (*Z*)-selectivity in the formation of 16-membered UM [62] and other lactones [48].

In 2013, Hoveyda proposed the use of dithiolate ligands to improve the (*Z*)-selectivity of RCM catalysts [93]; this work initiated the development of (*Z*)-selective catalysts of the formation of UMs. The Ru complexes containing the dithiocatechol fragment **Ru37** showed very low activity but unprecedented (*Z*)-selectivity in the RCM of CH_2_=CH(CH_2_)_7_COO(CH_2_)_5_CH=CH_2_ at high substrate concentrations (200 mM instead of the typically used 5 mM) [83]. When using dithiolate NHC catalysts (e.g., **Ru38**), the configuration of the C=C bond in UMs depends on the configurations of C=C bonds in starting alkenyl alkenoates; (*Z*)-isomers of 12–17-membered UMs were obtained from (*Z*)-alkenyl ω-alkenoates with a >95% stereoselectivity [84,94]. However, the presence of two (*E*)-alkenyl fragments in the substrates resulted in the formation of (*E*)-UMs during RCM catalyzed by **Ru39** and **Ru40** [85]. For the synthesis of (*Z*)-UMs using **Ru37**–**Ru40,** Hoveyda and coll. proposed an in situ methylene capping approach based on the addition of (*Z*)-but-2-ene to α,ω-dienyl substrate, with the formation of (*Z*)-MeCH=CH-terminated alkenyl alkenoate that was subsequently subjected to cyclization with high stereoselectivity; when (*E*)-but-2-ene was used, (*E*)-UMs were formed [86]. Among dithiolate Ru catalysts, the complex **Ru40** demonstrated the highest activity and stereoselectivity.

**Scheme 12:**
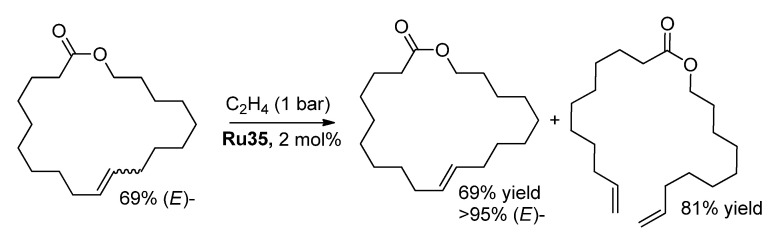
(*E*)-enrichment of the RCM products by (*Z*)-selective ethenolysis [48].

Note that the content of (*E*)-isomer in RCM products can be increased by (*Z*)-selective ethenolysis (Scheme 12) [48], but a similar approach cannot be considered as atom-efficient.

An alternative approach to the pure (*Z*)-isomers of unsaturated macrolactones was proposed by Fürstner and coll. [95]: the RCM of alkynyl alkynoates (20 mM in C_6_H_5_Cl) with the formation of 13- and 17-membered lactones (5 mol% Mo(CO)_6_/*p*-chlorophenol, 140 °C), with subsequent hydrogenation over Lindlar catalyst, resulted in the formation of Yuzu lactone and ambrettolide with high *Z*-selectivity.

At the end of this part of the review, mainly devoted to kinetically controlled RCM with the formation of UMs, some generalizations and conclusions are worth drawing.

First, the common dilution principle in the RCM of alkenyl alkenoates cannot be ignored. The rational design of metathesis catalysts allow one to obtain UMs with high isolated yields, but compliance with the dilution principle actualizes the problem of the use and utilization of the solvents. In most of earlier and even recent works, CH_2_Cl_2_ [30,40,49,50,52,54,55,83,84] or ClCH_2_CH_2_Cl [47,48,71] were used. However, because of the environmental issues, less toxic and dangerous solvents have been applied for RCM. In particular, relatively high conversions and yields were achieved in toluene [42,45,59,60,61,66,68,70,74,75,81,89], ethyl acetate [41,43,65,67,72,76,96], THF [85,86] and dimethyl carbonate [96]. The use of benzene as a solvent for RCM was proposed in a number of works [33,51,58,64,78] but seems not suitable for scaling due to the high toxicity of benzene. The traces of water deactivate catalysts [62], thus creating additional requirements for the solvent purity. Supercritical CO_2_, a popular green solvent, was also studied as a reaction medium for RCM [56], but the catalyst’s activities and UM yields were moderate. The problems of high dilution and solvent utilization in the synthesis of UMs have been solved by the use of high-boiling non-toxic hydrocarbons at elevated temperatures—but only for thermodynamically driven processes [31] (see Section 2.4.1 below).

Second, the availability of the catalytic component is critical in light of the possible application of RCM in the production of UMs. Even the use of a low catalyst loading does not remove the problem of the recycling, especially for more expensive Ru-based catalysts. The problems of the separation and recycling of Ru were discussed in an early review [97]; however, these problems are still relevant. Attempts to prepare heterogeneous recyclable catalysts for the RCM of alkenyl alkenoates have already been made (see Section 2.4.2 below), but the problem is far from being solved.

### 2.4. Ru-Catalyzed Ring-Closing Metathesis of Alkenyl Alkenoates at High Concentrations

#### 2.4.1. Ring-Closing Metathesis with Distillation of the Products

**Figure 4:**
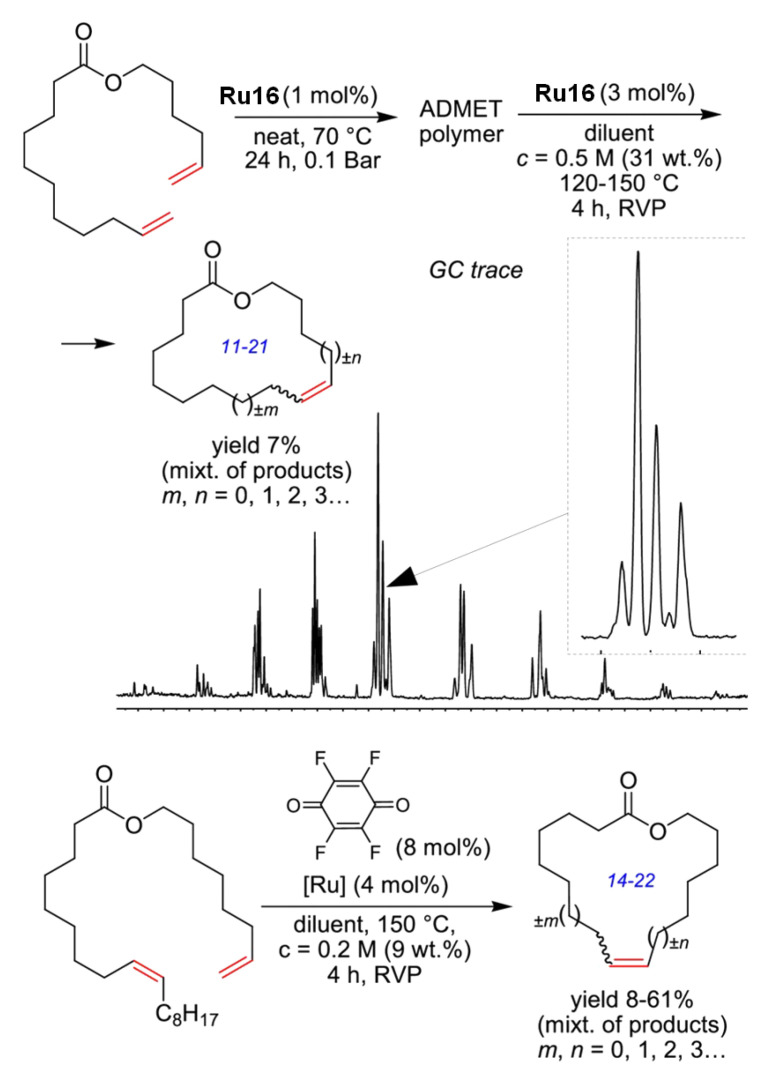
ADMET and then RCM depolymerization approach to macrolactones and GC trace of product. Reprinted with permission from [31]. Copyright (2018) American Chemical Society.

The key problems in RCM are related to the unfavorable entropic effect that makes the oligomerization (ADMET) dominate over the desired formation of Ums; moreover, the initial formation of oligomers was detected during RCM [49]. The ring–chain equilibrium is complicated by the formation of larger macrocycles (di-, tri-, tetramers, etc.) [98]. The desired shift of the UM/cyclic oligomer/polymer equilibrium can be achieved by the elimination of the UM from the reaction mixture. In 2018, Grela and coll. carried out the study of RCM at concentrations much higher than commonly used [31]. They proposed that the UMs can be removed from the reaction mixture by distillation in vacuo. The ADMET of hex-5-en-1-yl undec-10-enoate and subsequent depolymerization in the presence of **Ru16** (1 mol%) resulted in the formation of (*Z*/*E*) mixtures of 11–21-membered macrocycles with a maximum yield of 24% (Figure 4). The yields were increased after the addition of TFQ, but C=C bond migration and the formation of MUs of different sizes were still observed.

**Scheme 13:**
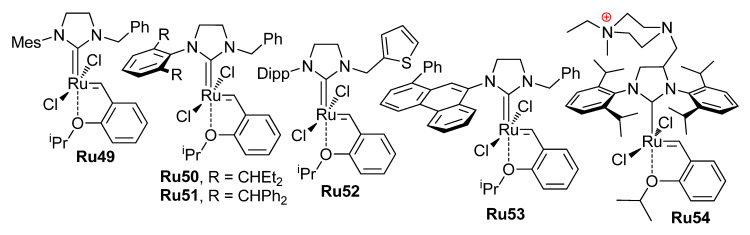
NHC Ru catalysts studied in RCM of alkenyl alkenoates with distillation of UMs [31].

**Figure 5:**
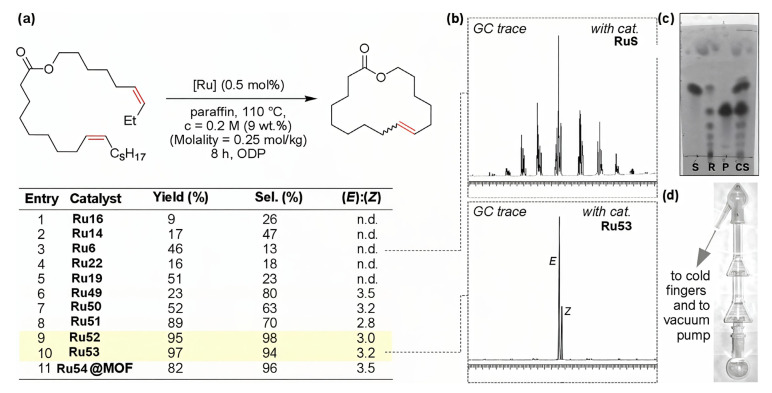
RCM experiments: (**a**) Yield and selectivity of the formation of macrolactone (Selectivity = (Int. of *E* + *Z*)/(Int. of all products); the most efficient catalysts are marked in yellow. (**b**) GC traces of products obtained using complexes **Ru6** and **Ru53**. (**c**) TLC analyses of reaction mixture from experiment ceased after 20 min. S—standard of the substrate, R—sample of reaction mixture, P—standard of the product, CS—all three together. (**d**) Example of a reaction/distillation Hickman glass apparatus. Reprinted with permission from [31]. Copyright (2018) American Chemical Society.

A comparative study of Ru complexes **Ru6**, **Ru14**, **Ru16**, **Ru19**, **Ru22** (Scheme 6) and **Ru49**–**Ru54** (Scheme 13) was conducted in experiments in paraffin oil at 110 °C (10^−6^ mBar); the substrate concentration was 0.2 M. The results of the experiments were qualitatively dependent on the type of the catalyst used (Figure 5) [31].

Note that under homogeneous conditions, **Ru54** showed a low selectivity in the cyclization of CH_2_=CH(CH_2_)_8_COO(CH_2_)_4_CH=CH_2_ that was substantially improved when this catalyst was immobilized on a metal–organic framework (MOF) [67]. This heterogeneous catalyst was used in experiments with the distillation of the UMs in [31].

A number of alkenyl oleates were transformed to 13-, 16-, 17- and 19-membered unsaturated lactones; the yields were 60–93%, and the (*E*)/(*Z*) ratio was 2.1–4.9 depending on the ring size (Table 4); the esters of dec-9-enoic acid demonstrated similar results.

Another example of the synthesis of UM with its distillation is presented in [83]: in the presence of **Ru38** (10 mol%), the cyclization of (*Z*)-non-6-enyl oleate (200 mM in PAO6, 130 °C, 8 h) resulted in the formation of 16-membered UM with an 80% yield and 78% selectivity and a (*Z*)/(*E*) ratio of 74:26; **Ru41** under similar conditions catalyzed the formation of UM with a 31% yield and >99% (*Z*)-selectivity.

**Scheme 14:**
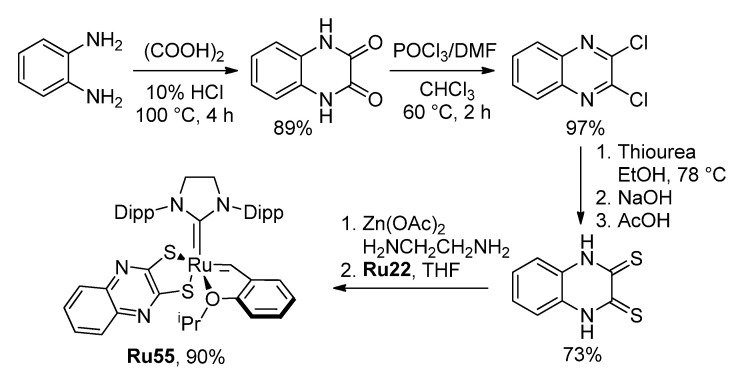
Preparation of the catalyst **Ru55** [99].

**Figure 6:**
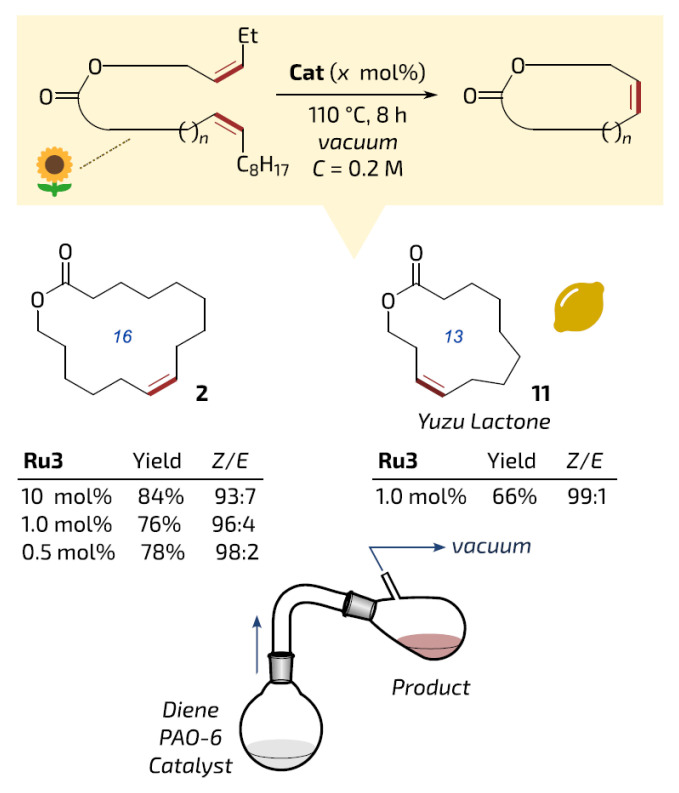
Examples of bio-based (partially or fully derived from oleic acid, which is symbolized by sunflower icon) macrocycles obtained in reactive vacuum distillation at high concentration. Yields of isolated pure products. Reprinted with permission from [99]. Copyright (2024) Springer Nature.

Very recently, Grela and coll. synthesized the highly prospective catalyst **Ru55** for high-temperature RCM [99]. The synthesis of **Ru55** was based on a cheap ligand precursor, prepared from benzene-1,2-diamine in five simple steps, and **Ru22** (Scheme 14). The high efficiency of **Ru55** in (*Z*)-selective RCM with distillation of the reaction products was demonstrated in examples of the synthesis of 16- and 13-membered UMs (Figure 6).

#### 2.4.2. Ring-Closing Metathesis Under Spatial Confinement Conditions

**Scheme 15:**
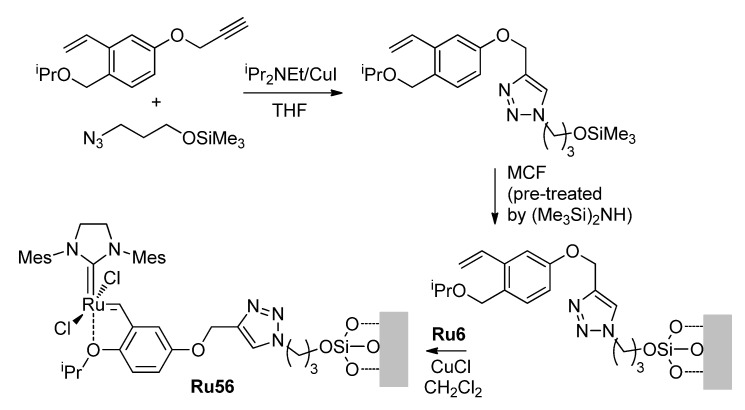
Synthesis of silica-immobilized catalyst **Ru56** [101].

In 2013, Lee, Hong and co-workers reported the results of their study of the HG-II-type catalyst **Ru56** immobilized on mesocellular siliceous foam (MCF) bearing large nanopores (Scheme 15). A comparative study of **Ru14** and **Ru56a**–**Ru56c** (**Ru56** immobilized on MCF with average pore diameters of 24.1 nm (**a**) and 20.6 nm (**b**) or on non-porous SiO_2_ (**c**)) revealed the higher activity of the MCF-immobilized catalysts **Ru56a** and **Ru56b** in the RCM of CH_2_=CH(CH_2_)_8_COO(CH_2_)_n_CH=CH_2_ (*n* = 2, 4, 8, 9) in comparison with **Ru14** [100] (Table 5). And most importantly, when concentrations of the substrates were increased to 50 mM, the yields of UMs amounted to 4–7% when using **Ru14** and 42–55% in the presence of **Ru56a**. In 2019, Grela and coll. studied the RCM of hex-5-en-1-yl undec-10-enoate in the presence of **Ru54**, noncovalently immobilized in sulfated MOF; a yield of 72% was achieved at a 5 mM concentration of the substrate [96].

**Scheme 16:**
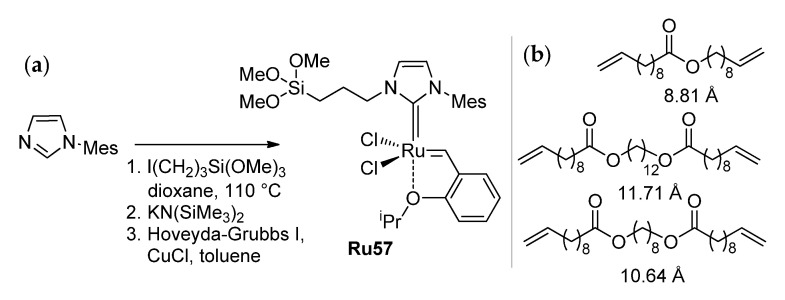
(**a**) Synthesis of **Ru57** catalyst capable of immobilization on silica. (**b**) Structures and hydrodynamic diameters (DOSY-NMR data) of RCM substrates [102].

**Figure 7:**
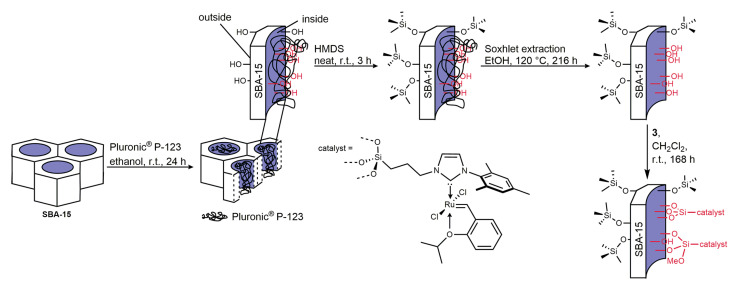
Multistep modification of SBA-15 for pore-selective immobilization of **Ru57** inside mesopores [102]. Copyright (2019) American Chemical Society.

In 2019, Buchmeiser and coll. proposed an alternative route to solve the problem of the substrate concentration by RCM in confined geometries, using an olefin metathesis catalyst (**Ru57**, Scheme 16a) selectively immobilized inside ordered mesoporous silicas SBA-15 (Figure 7) with pore diameters *d* of 50 and 62 Å [102]. It was shown that the selectivity of macrocyclization substantially increases with an increase in the hydrodynamic diameter of the substrate (Scheme 16b) when using an SBA-15_50_-based catalyst: e.g., dodecane-1,12-diol-based diester formed up to 60% of the RCM product at a 25 mM concentration. For dec-9-en-1-yl undec-10-enoate (25 mM in C_6_D_6_) in the presence of 0.1 mol% of the catalyst, the MU/oligomer ratio doubled in comparison with the MU/oligomer ratio achieved under homogeneous conditions.

The further studies of **Ru57**, immobilized on mesoporous silica, in the RCM of CH_2_=CH(CH_2_)_8_COO(CH_2_)_8_CH=CH_2_ (25 mM in toluene) [103,104] showed that, during macrocyclization under confinement, ADMET processes are limited by the formation of short-chain oligomers (dimers and trimers), which in turn shifts the overall ring–chain equilibrium towards the desired direction and thus increases the selectivity of UM formation.

**Figure 8:**
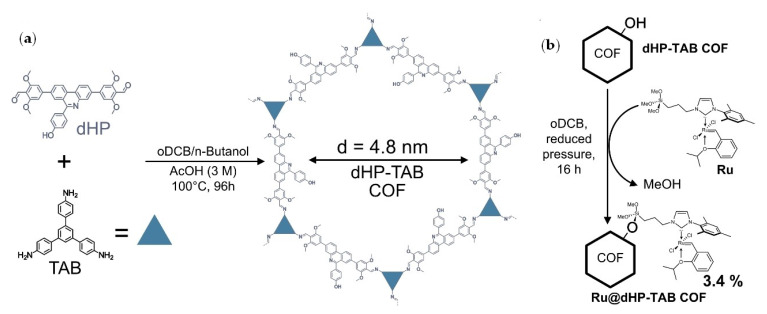
(**a**) Synthesis of covalent organic framework (COF). (**b**) Immobilization of **Ru57** on COF by silylation. Reprinted with permission from [105]. Copyright (2022) Wiley-VCH Verlag GmbH and Co.

In 2022, Lotsch, Buchmeiser and coll. developed **Ru57**, immobilized on a covalent organic framework (COF, Figure 8) [105]. For CH_2_=CH(CH_2_)_8_COO(CH_2_)_8_CH=CH_2_, an increase of 51% in the UM/oligomers ratio from 0.90 for **Ru57** to 1.35 for immobilized **Ru57**/COF was found, corresponding to a 59% increase in selectivity compared to the homogeneous catalyst.

### 2.5. Ring-Closing Metathesis Using Group 6 Metal Complexes

Historically, the first publication devoted to the use of RCM in the synthesis of unsaturated macrolactones (Villemin, 1980) describes experiments on the cyclization of CH_2_=CH(CH_2_)_8_COO(CH_2_)_n_CH=CH_2_ (*n* = 3, 4; 15 mM in C_6_H_5_Cl at 75 °C) in which WCl_6_/Me_4_Sn (5 mol%) was used as a catalyst; the yields of 15- and 16-membered lactones were 60 and 65%, respectively [14]. One year later, Tsuji and Hashiguchi studied the RCM of oleyl oleate and CH_2_=CH(CH_2_)_8_COO(CH_2_)_9_CH=CH_2_, catalyzed by WCl_6_ (or WOCl_2_)/(η^5^-C_5_H_5_)_2_TiMe_2_; the yields did not exceed 18% [106].

**Scheme 17:**
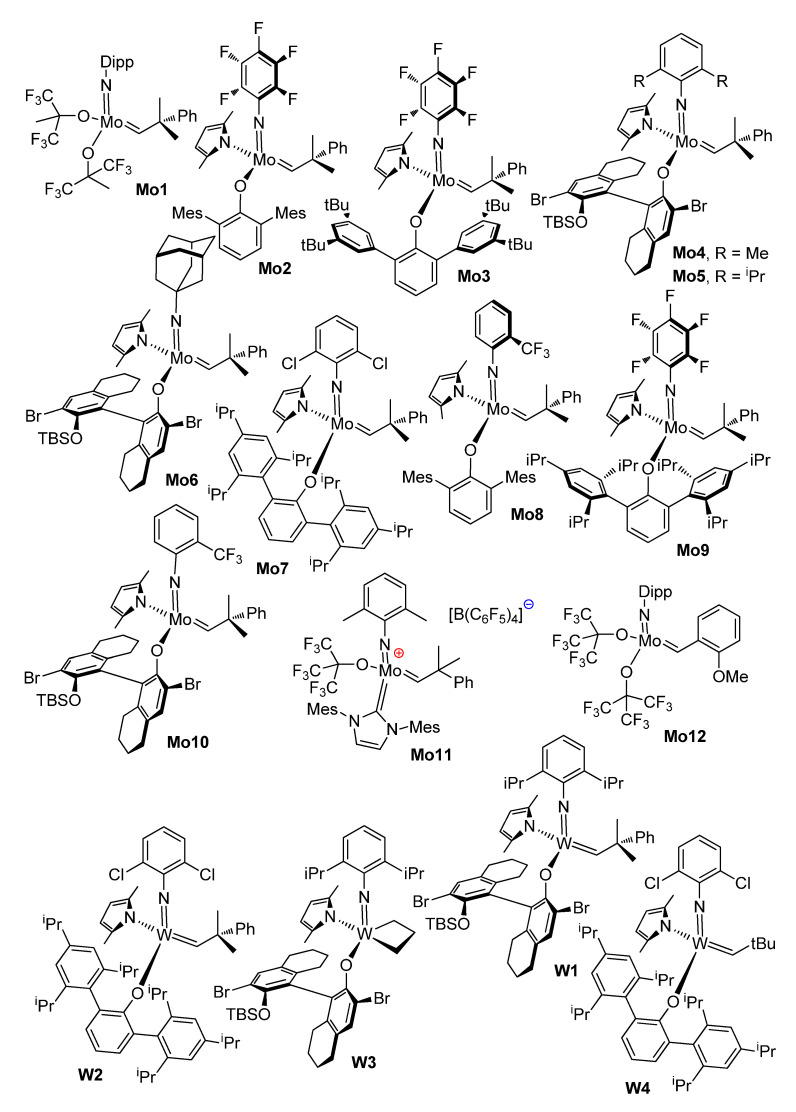
Group 6 metal complexes studied in RCM of alkenyl alkenoates.

Highly active Shrock catalysts, which represent Mo and W carbene complexes supported with imido and alkoxy (aryloxy) ligands [107] (see Scheme 17), were studied in the RCM of alkenyl alkenoates in the 2010–2020s.

In 2011, Hoveyda and coll. reported the results of comparative studies of **Ru6** and Group 6 metal complexes **Mo1**, **Mo4**, **Mo5**, **Mo6**, **W1** and **W4** (Scheme 17) in the RCM of CH_2_=CH(CH_2_)_3_COO(CH_2_)_9_CH=CH_2_ with the formation of 16-membered UM [45]. As can be seen in Table 6, **Ru6**, **Mo1** and **Mo4** showed moderate activity and stereoselectivity, whereas **Mo5**, **W1** and **W4** turned out to be (*Z*)-selective catalysts. The complexes **Mo2** and **Mo3** showed moderate activities in the RCM of alkenyl alkenoates containing trisubstituted olefinic fragments [108].

The stereoselectivity of **Mo6** depended on the size of the UM: for 5 mM substrate solutions in toluene at 22 °C with a 3 mol% catalyst loading, the (*Z*)-selectivity was 69, 80, 85 and 91% for 13-, 14-, 16- and 17-membered macrolactones, respectively [46]. The complex **W3** (5 mol%) showed a similar productivity and 73, 82 and 88% (*Z*)-selectivities for 13, 14 and 17-membered UMs. The isolated yields of Yuzu lactone (13-membered) and ambrettolide (17-membered) were ~50 and 80%, respectively. It was also demonstrated that W catalysts are less sensitive to oxygen and moisture in comparison with Mo complexes [46].

**Scheme 18:**
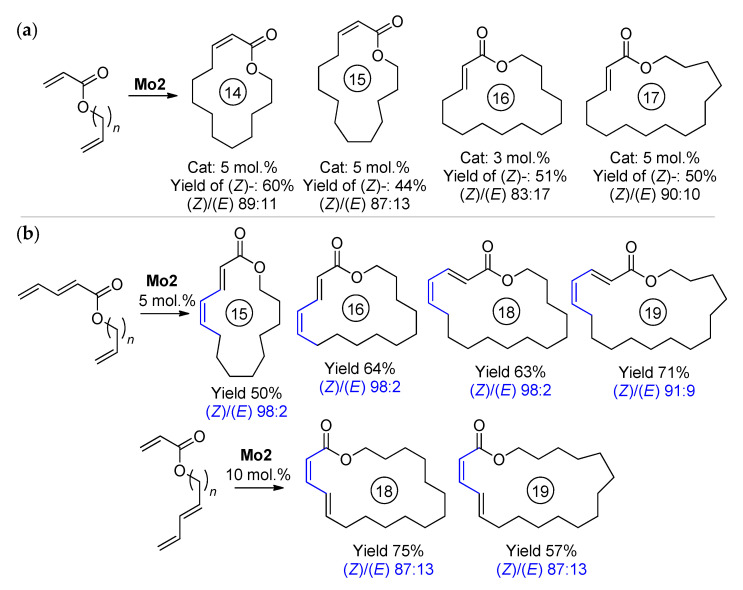
(*Z*)-selective RCM, catalyzed by **Mo2**: (**a**) Cycllization of acrylates. (**b**) Cyclization affording (*E*,*Z*)- and (*Z*,*E*)-dienoates. Substrate concentration 2 mM in C_6_H_6_, 22 °C, 100 torr [78].

In 2014, Schrock and Hoveyda for the first time successfully carried out the RCM of ω-alkenyl acrylates [78]. In the RCM of CH_2_=CHCOO(CH_2_)_10_CH=CH_2_ **Ru14** showed moderate activity (see Table 3); **Mo4**, **Mo8**, **Mo9** and **W4** showed low conversions (˂10% after 2 h at 10 mol% catalyst loading); **Mo1** and **Mo10** were moderately active (30 and 61%, respectively); and the complex **Mo2** showed a 60% yield and 89% (*Z*)-selectivity. Other ω-alkenyl acrylates and unsaturated esters, containing a conjugated diene fragment, were also converted to UMs using **Mo2** (Scheme 18).

**Figure 9:**
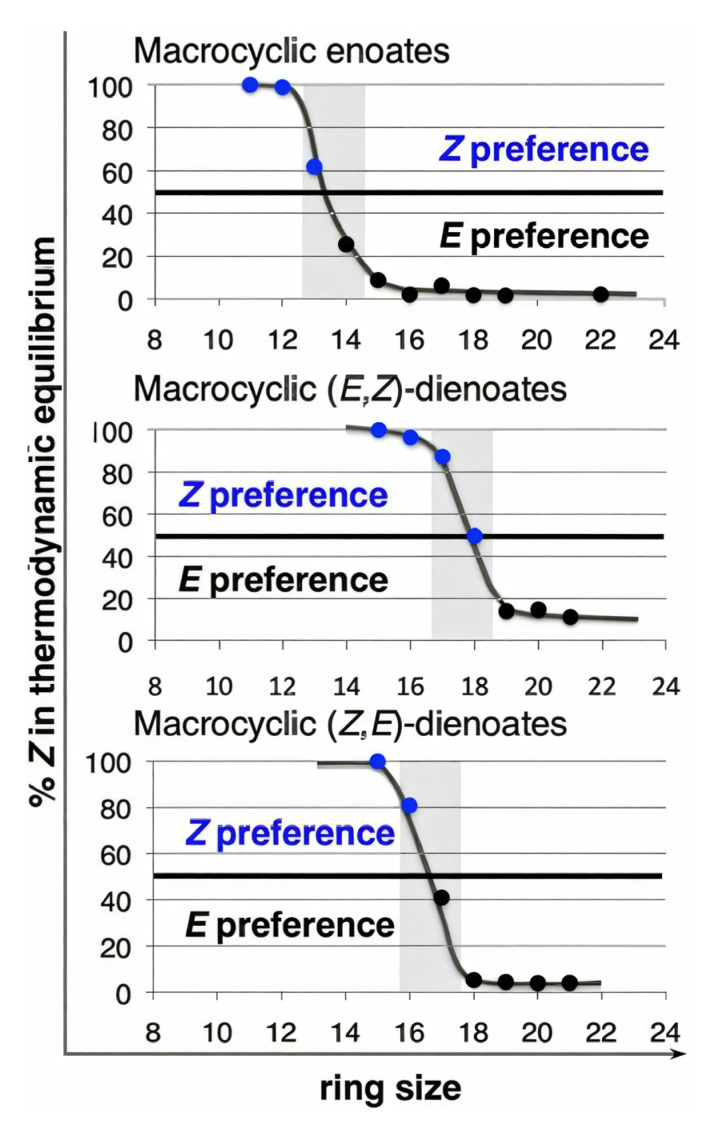
Variations in the thermodynamic preferences of different unsaturated macrocyclic alkenes. Reprinted with permission from [78]. Copyright (2014) American Chemical Society.

The results of DFT calculations of the relative stability of (*Z*)- and (*E*)-isomers of MUs with one C=C bond conjugated with a C=O group (macrocyclic enoates) and conjugated isomeric dienoates are presented in Figure 9 [78]. For enoates and dienoates, the thermodynamic preference for the (*E*)-isomer is reached at large (14+) ring sizes; therefore, the composition of the reaction mixtures, formed with the use of the **Mo2** catalyst, is determined by kinetic control—in other words, by the (*Z*)-stereoselectivity of the **Mo2** catalyst. The kinetically controlled (*E*)-selective synthesis of UMs can be provided by the use of diene substrates with (*E*)-configurations of C=C bonds. In 2017, Hoveyda and coll. investigated the catalytic behavior of **Mo3** (5 mol.%, 22 °C, 2 h at 28 torr) in the RCM of (*E*)-CH_2_=CH(CH_2_)_3_COO(CH_2_)_9_CH=CHX (1 mM in toluene) [109]. High (*E*)-selectivities (96 and 93%) were achieved when using substrates containing X = –B(OCMe_2_CMe_2_O) (B(pin)) and –B(OCMe_2_CH_2_CHMeO), respectively. The RCM of functionalized substrates (*E*)-CH_2_=CH(CH_2_)_n_COO(CH_2_)_m_CH=CHB(pin) under the same conditions resulted in the formation of cyclic lactones; the yields and (*Z*)/(*E*) ratios were 60% and 4:96 (*n* = 3, *m* = 9), 57% and 4:96 (*n* = 5, *m* = 3), 40% and 9:91 (*n* = 6, *m* = 3), 40% and 6:94 (*n* = 6, *m* = 4), 59% and 2:98 (*n* = 8, *m* = 4) and 40% and 9:91 (*n* = 6, *m* = 9). A 21-membered lactone (2:98 (*Z*)/(*E*) ratio) was synthesized with a 51% yield from a B(pin)-functionalized derivative of unced-10-enoic acid B(pin)CH=CH9CH_2_)_8_COO(CH_2_)_9_CH=CH_2_. The synthesis of 12-membered UM from CH_2_=CH(CH_2_)_6_COO(CH_2_)_2_CH=CHB(pin) deserves special mention: a 48% yield of pure (*E*)-isomer was achieved (when using an **Ru2** catalyst, the maximum yield was 29% with a (*Z*)/(*E*) ratio of 21:79).

The idea of the use of the ordered mesoporous support SBA-15 (RCM in confined geometries) was also implemented for Mo complexes of *N*-heterocyclic carbenes, e.g., **Mo11 [110]**. Up to 98% RCM selectivities were achieved at a 1 mol% loading of the catalyst and up to a 100 mM concentration of the substrates. However, **Mo11** is cationic, which imposes certain restrictions on its use due to a lower chemical stability and higher sensitivity to catalytic poisons.

**Figure 10:**
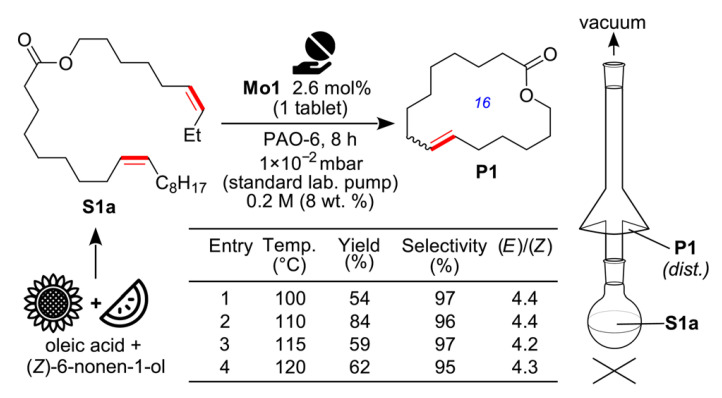
Practical promise of high-concentration RCM promoted by **Mo1** in synthesis of 16-membered UM. Reprinted with permission from [111]. Copyright (2023) Royal Society of Chemistry.

Perhaps the most interesting actual study in the field of the RCM of alkenyl alkenoates, catalyzed by Mo complexes, was performed by Grela and coll. [111]. The high thermal stability of metathesis catalyst **Mo1** was successfully implemented in the vacuum distillation RCM of (*Z*)-non-6-en-1-yl oleate (Figure 10).

**Scheme 19:**
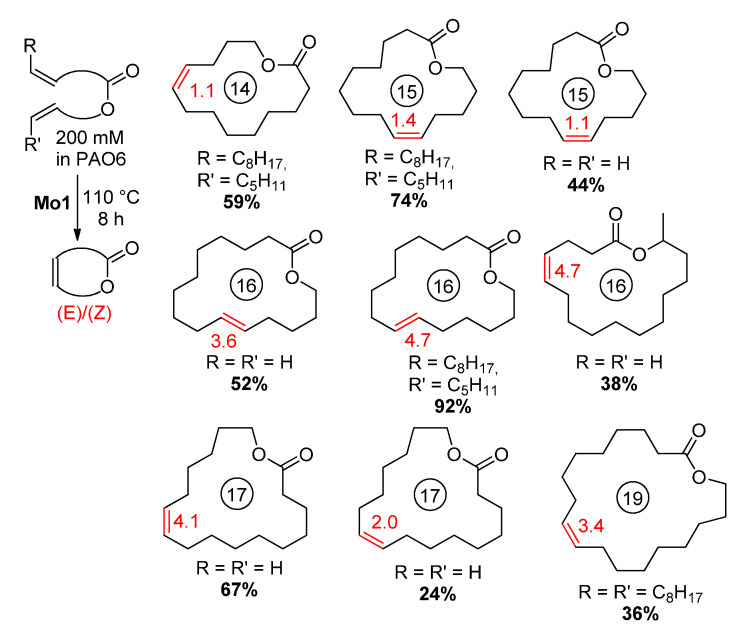
Scope and limitations of high-concentration RCM promoted by **Mo1 [111]**.

Since molybdenum alkylidenes did not cause a C=C migration even at 100–120 °C, the reaction proceeded with high selectivity, leading to well-defined UMs (Scheme 19).

### 2.6. Ring-Closing Depolymerization of Unsaturated Polyesters

The results of a number of the above-mentioned works can be considered as examples of the depolymerization approach to UMs—the actual processes of the RCM of alkenyl alkenoates [31,83,99,111], based on the distillation of low-MW UMs, involve the intermediate formation of the unsaturated oligo-/polyesters. Ring-closing depolymerization (cyclodepolymerization, CDP) deserves special consideration in view of the actual trends in polymer chemistry, focused on polymer recycling and/or upcycling [112,113,114], in the framework of the circular plastics economy concept [115,116]. CDP with the formation of UMs is a special case of metathesis polymer deconstruction described in a recent review of Wang and coll. [117].

Metathesis CDP, as well as the RCM of alkenyl alkenoates, requires high dilutions (mM concentrations of the macrocycles formed) and near quantitative conversions [9]. As mentioned above, thermodynamically controlled CDP may be implemented via the elimination of the low-MW UMs from the reaction mixtures at elevated temperatures. However, biasing the kinetic product distribution toward the formation of macrocycles can also be realized, in particular by the development of new catalysts [118].

The synthesis of UMs via kinetically controlled CDP has not been sufficiently examined so far. Some key patterns of similar processes were revealed very recently by Foster and coll. by an example of isomeric bis(ω-alkenyl) phthalates and the corresponding ADMET and RCM products with the use of **Ru2**, **Ru6**, **Ru13**–**Ru15** and **Ru17** [119]. The catalyst **Ru17** showed the most promising characteristics, enabling the reaching of high conversions of polymers to RCM products at substrate concentrations up to 0.1 M. Further studies as applied to the synthesis of aliphatic UMs hold promise.

### 2.7. Transesterification Depolymerization of Unsaturated Polyesters

**Scheme 20:**
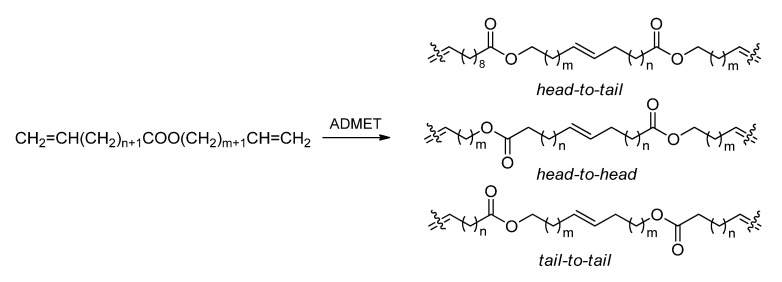
The structural fragments in the products of the ADMET of alkenyl alkenoates.

As was mentioned above, it has been proven in a number of cases [33,49] that UMs are formed from low-MW oligomers, the products of the ADMET of alkenyl alkenoates via metathesis backbiting. However, the backbiting of ADMET products can also be carried out through transesterification, via the intramolecular cleavage of C(O)–O bonds in copolymer. The natural limitation of this alternative approach is the statistical nature of the ADMET products that evidently include head-to-tail, head-to-head and tail-to-tail units in their structures (Scheme 20). A similar statisticity does not matter for RCM, but it complicates ring-closing transesterification significantly.

During the transesterification of CH_2_=CH(CH_2_)_7_COO(CH_2_)_6_CH=CH_2_ polymer with the distillation of the reaction products (PEG200 as a solvent, 190–200 °C, 10 mbar, 40 h, Ti(O^i^Pr)_4_), a 75% yield of ambrettolide was achieved (43 g of the product of 87% purity from 100 g of polymer) [120]. The yield in the experiment on metathesis depolymerization with distillation, catalyzed by **Mo12**, was 60% [120]. The depolymerization of polyester, obtained by the ROTEP of ω-6-hexadecenlactone (see Section 3.1), with the use of ZnCl_2_/PEG600, recovered 40% of the starting monomer [121].

Summarizing the information presented in Section 2 in view of the potential industrial scalability and sustainability of the RCM of alkenyl alkenoates, let us dare to propose most promising directions for the further development of this catalytic process according to Green Chemistry principles [122] and actual standards for advanced chemical technologies.

- To avoid the problems associated with the use of large volumes of solvents (and related problems of toxicity, recycling, etc.), the further development of the Grela approach, based on the distillation of low-MW target products, appears to be a promising technical solution. The use of non-toxic [123] hydrogenated dec-1-ene oligomers as a reaction medium is an added benefit of this approach;
- It makes sense to guide the further design and development of the catalysts toward thermally stable heterogeneous systems. It is not obvious that Ru-based catalysts are out of the competition: Mo-based catalysts also demonstrated promising results [111]; further studies make perfect sense;
- In terms of circular economy, the RCM or ring-closing transesterification of unsaturated polyesters might be a substantial source of UMs and related cyclic substrates for subsequent polymerization (see Section 3).

However, these efforts are meaningful if the UMs will be in demand and cannot be considered without taking into account the prospects of the use of UMs for the development of new materials (see Section 3 below).

## 3. Polyesters Based on Unsaturated Macrolactones

In Section 2, the preparation of a wide range of unsaturated macrolactones was described. Many of these compounds—at least in theory—can be considered as a substrates for the synthesis of unsaturated polyesters. Evidently, there are two different catalytic approaches to similar polymers [2].

**Scheme 21:**
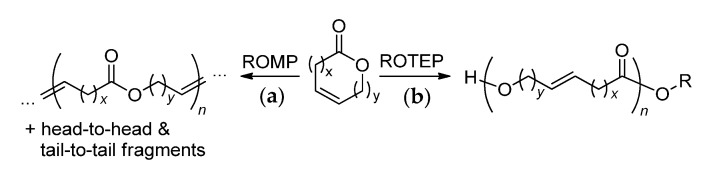
Two main synthetic approaches to unsaturated polyesters: (**a**) ring-opening metathesis polymerization and (**b**) ring-opening transesterification polymerization.

The first is based on ring-opening metathesis polymerization (Scheme 21a). The significant drawbacks of this approach are as follows:

- Low regioselectivity: formation of head-to-tail, head-to-head and tail-to-tail fragments (see Scheme 20);
- Excessive complexity: the same polymers can be obtained by the ADMET of alkenyl alkenoates, avoiding the labor-intensive stage of the synthesis of UMs.

The second approach is ring-opening transesterification polymerization (ROTEP, Scheme 21b). Being initiated by ROH in the presence of the catalyst of complex metal alkoxide L_n_MOR, ROTEP results in the formation of polyesters containing only head-to-tail fragments. The polycondensation of ω-hydroxyalkenoic acids results in similar products, but ROTEP is usually preferred as a less energy-consuming method that provides a higher level of chain control (the degree of polymerization and dispersity Ð_M_).

Polymers based on macrolactones were the subject of the brief review of Dove, Heise and coll., published in 2019 [2]. In this review, significant attention has been given to (co)polymers of saturated macrolactones; however, the main features and specifics of the synthesis of polyesters and the prospects of their applications have been insufficiently addressed. In this section, we tried to summarize all the information concerning UM-based (co)polymers, which has not been done previously.

### 3.1. Ring-Opening Transesterification Polymerization of Unsaturated Macrolactones

**Scheme 22:**
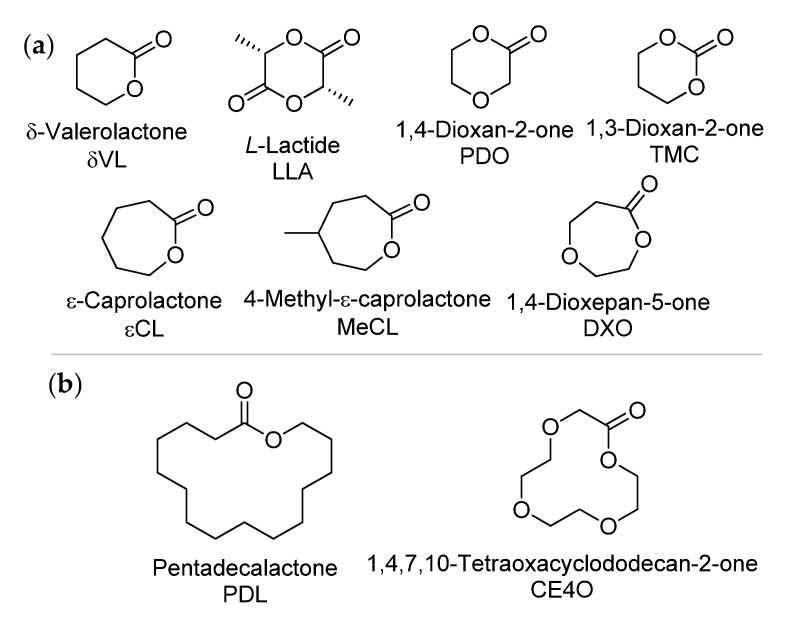
Conventional ROTEP substrates: (**a**) Strained cyclic esters. (**b**) Saturated macrolactones.

**Scheme 23:**
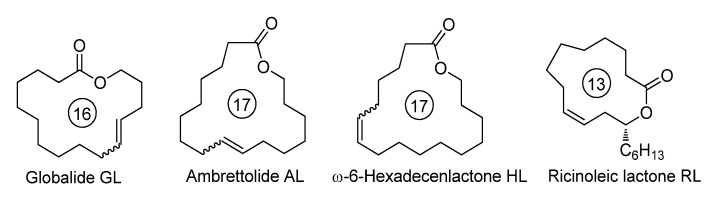
Unsaturated macrolactones used in ROTEP as cyclic monomers.

As mentioned above (and widely accepted in the scientific community [124,125]), the ROTEP of lactones provides excellent control over the polymerization process to produce high-MW products with a sharp molecular weight distribution under relatively mild reaction conditions. There is a qualitative difference between the ROTEP of medium ring-sized cyclic esters (6-membered δ-valerolactone δVL, lactides LA, 1,4-dioxan-2-one PDO, 1,3-dioxan-2-one (trimethylene carbonate) TMC, 7-membered ε-caprolactone εCL, 1,4-dioxepan-5-one, etc., see Scheme 22) and macrolactones (including saturated monomers, see Scheme 22 and Scheme 23). In the first case, the process is driven by ring strain, but in the latter case ROTEP is more entropically driven [126]. For entropically driven polymerization, the advantages of enzymatic catalysis are long known [127], and it was this type of catalyst that was used for the ROTEP of UMs in most works (Section 3.1.1). In addition, the use of coordination catalysts in the ROTEP of UMs is also known and presented in a number of examples (Section 3.1.2).

In this part of the review, we tried to summarize and discuss all the published information related to the ROTEP of UMs. Surprising but true: despite the diversity of UMs separated and/or synthesized to date, only four UMs have been investigated as a ROTEP substrate, namely globalide (GL), ambrettolide (AL), isomeric to the latter ω-6-hexadecenlactone (HL), and the product of the intramolecular esterification of ricinoleic acid (RA), the corresponding 13-membered substituted lactone (RL, Scheme 23).

#### 3.1.1. Enzyme-Catalyzed ROTEP and Copolymerization of UMs

Certain aspects of the lipase-catalyzed ROTEP of UMs were discussed in a recent review (2023) devoted to the polymerization of natural compound-based cyclic monomers [128]. Below, we summarized the results of the studies of the (co)polymerization of UMs with the use of enzymatic catalysis. To date, lipase family enzymes (mainly *Candida antarctica* Lipase B) have been investigated in the ROTEP of Ums; the polymer yield and characteristics were significantly dependent on the type of cyclic substrate, the presence and amount of comonomer and catalyst and reaction conditions [129,130,131,132,133,134,135,136,137,138,139,140,141,142,143,144,145] (Table 7).

The homopolymerization of AL and HL, catalyzed by Novozym 435 (the commercially available catalyst, *Candida antarctica* Lipase B, immobilized on cross-linked polyacrylate) was first performed in 2008 [129]; the semicrystalline polymers had *T*_m_ = 58.8 and 96.2 °C (first heating run) and crystallization temperatures *T*_c_ = 37.7 and 76.6 °C, respectively. Over the years, the methods of the (co)polymerization of UMs have been worked out (Table 7); copolymers with εCL, MeCL, DXO, CE4O and PDL were prepared. Ther mini-emulsion polymerization of GL using pure lipase resulted in the formation of nanoparticles with an average diameter *d* = 80–280 nm [135]. In 2018 [137], it was shown that the commercial catalyst NS 88011 demonstrates higher activities in the ROTEP of GL in comparison with Novozym 435. The Novozym 435-catalyzed ROTEP of GL and GL/PDL copolymerization were optimized recently with the use of supercritical CO_2_ or propane reaction medium [141,146,147,148]. The reversibility of the enzyme-catalyzed ROTEP of GL and HL was demonstrated recently by Martínez de Ilarduya and coll., setting the stage for the further development of the enzymatic recycling of similar polymers [149].

The Novozym 435-catalyzed ROTEP of GL, initiated by HO(CH_2_)_4_OH, resulted in the obtaining of well-defined macroinitiator that was used in the synthesis of a triblock copolymer with LLA, using Sn(Oct)_2_ as a catalyst [150].

#### 3.1.2. ROTEP and Copolymerization of UMs, Catalyzed by Acids and Metal Complexes

**Scheme 24:**
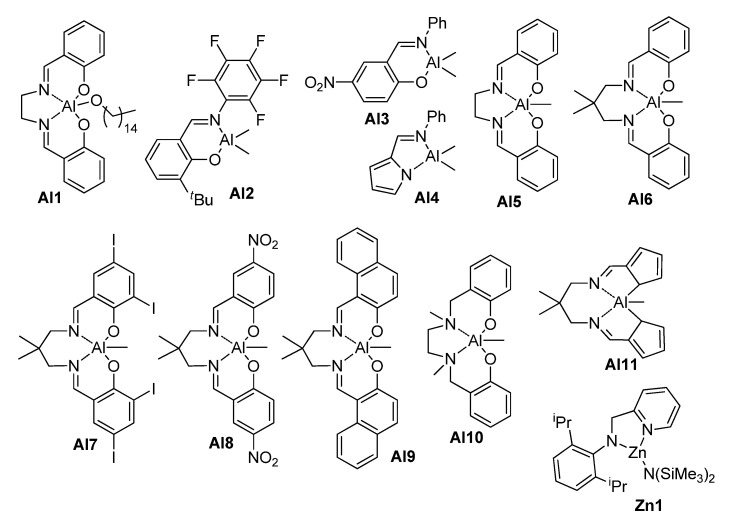
The structures of coordination catalysts studied in ROTEP of UMs.

The examples of the non-enzymatic ROTEP of UMs reported in scientific periodicals are relatively few in number [151,152,153,154,155,156,157] (Table 8); the structures of the catalysts are presented in Scheme 24.

In 2013, Mecerreyes and coll. reported the results of their study of the acid-catalyzed polymerization of GL and AL with the use of BnOH as an initiator [151]. The rate of polymerization depended on the p*K*_a_ value (CF_3_SO_3_H > Dodecylbenzenesulfonic acid (DBSA) > (PhO)_2_P(O)OH). In addition to bulk polymerization, mini-emulsion ROTEP was carried out, with the formation of latexes suitable for subsequent cross-linking with the use of benzoyl peroxide (BPO) [151].

The complex **Al1** (Scheme 24) was studied in the ROTEP of AL; homopolymers were characterized by a broadened MWD due to the partial formation of cyclic polymers with lower molecular weights [152]. One year later, the **Al1**-catalyzed synthesis of AL homopolymers and copolymers with PDL was described; the obtained (co)polymers had an *M*_w_ = 106–199 kDa and Ð_M_ = 2.2–2.7 [153]. The less sterically hindered complex **Al2** was used in the polymerization of HL [154]; homopolymers with an *M*_n_ = 8.9–36.3 kDa were obtained. This catalyst was also used for the synthesis of block- and stat-copolymers of HL with εCL [154,155]. In 2020, Naddeo et al. proposed the use of the pyridylamido zinc(II) complex **Zn1** (Scheme 24) as a catalyst of the BnOH-initiated ROTEP of HL and its copolymerization with εCL and PDL [156]. Poly(HL) had a *T*_m_ = 55 °C. Poly(HL-*co*-εCL) containing 73 mol% of εCL units had a *T*_m_ = 42 °C. Very recently, Al complexes **Al3**–**Al12** were synthesized and studied in the (co)polymerization of HL; the complexes **Al5**, **Al6** and **Al11** (Scheme 24) demonstrated higher activities [158].

The homopolymerization of RL, catalyzed by Me_3_SiONa, yielded low-MW polymer; the same results were obtained when using a mixture of RL with cyclic dimer and trimer [157]. In the same work, the copolymerization of RL with LLA, catalyzed by Tin(II) 2-ethylhexanoate (Sn(Oct)_2_), resulted in the formation of random copolymers containing 6.3–17 wt% of RA (melting point *T*_m_ = 105–130 °C).

### 3.2. Alternative Approaches to Unsaturated Polyesters

**Scheme 25:**
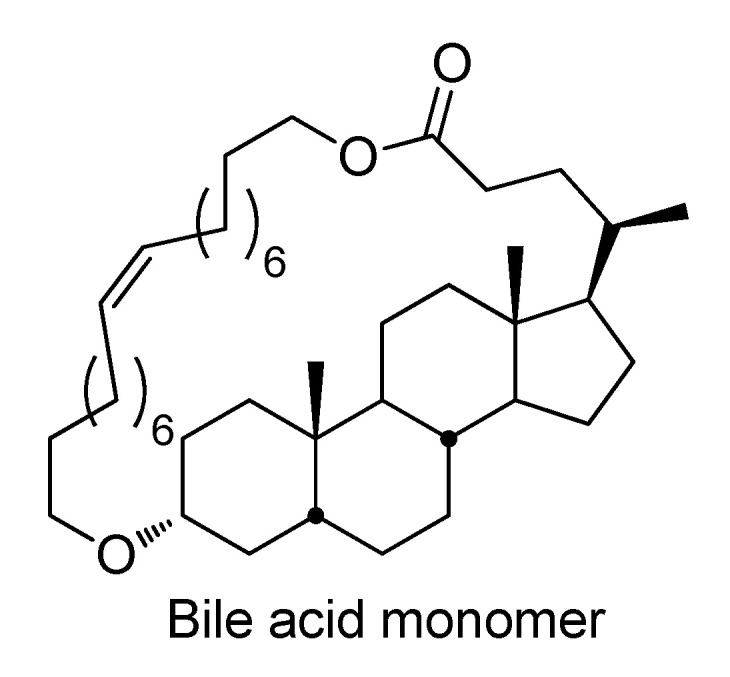
The structure of bile acid monomer used in co-ROMP with RL [164].

Evidently, UMs may also be subjected to ring-opening metathesis polymerization (ROMP), with the formation of polymers with a similar structure. GL and its homologs remain virtually unexplored in ROMP, because alkenyl alkenoates can be introduced to ADMET with the same results. However, if there is an efficient approach to UMs that does not use non-conjugated dienes as a raw materials, the use of ROMP makes more sense. The classic example of a similar synthetically available macrolactone is RL.

(Co)polymers of RA could be obtained by the polycondensation of RA or RA esters [159,160,161,162,163] and by the ROTEP [157] or ROMP [164,165] of RL. As was mentioned in Section 3.1, the ROTEP of RL was found to be ineffective in the synthesis of high-MW poly(RA). In 2020, hetero-dimeric monomers of RA and 4-hydroxycinnamic acid were prepared and introduced to polycondensation in the presence of *N*,*N*-dimethyleminopyridine (DMAP) or its tosylate; the highest *M*_n_ was 24.3 kDa [160]. Recently, high-MW poly(RA) was synthesized via the solution polycondensation of methyl ricinoleate in 1-alkyl-3-methylimidazolium bis(trifluoromethanesulfonyl)imide ILs with the retaining of a (*Z*)-configuration; homopolymers with an *M*_n_ up to 64.7 kDa were obtained [163]. Poly(RA) had a *T*_g_ = −72.8 °C and decomposition temperature *T*_d_ = 319 °C; the authors considered poly(RA) as a degradable elastomer. Catalytic ROMP (**Ru6**) was successfully applied in the synthesis of the copolymer of RA with bile acid monomer (Scheme 25); copolymers with an *M*_w_ up to 530 kDa were obtained [164].

**Scheme 26:**
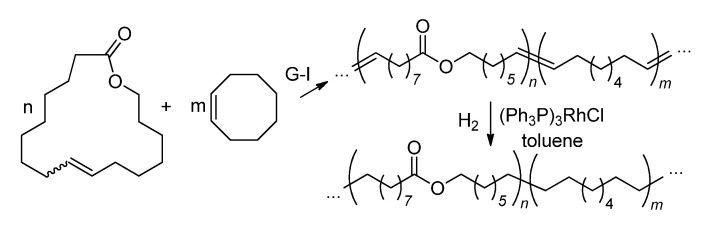
Ring-opening metathesis polymerization of AL and (*Z*)-cyclooctene and subsequent hydrogenation [166].

The ROMP of UMs starts to make sense when synthetically available cycloolefin is introduced into the copolymer backbone at a high ratio. In 2012, Duchateau and coll. synthesized a series of copolymers of AL and (*Z*)-cyclooctene (Scheme 26), used for the preparation of polar analogs of polyethylene (PE, see Section 3.3.3) [166]. The copolymerization of HL and norbornene in the presence of **Ru2**, **Ru6** or **Ru14** resulted in copolymers with an *M*_n_ up to 134 kDa; their elasticity increased with the increase in HL content [167]. The homopolymerization of HL (**Ru2**, **Ru6** or **Ru14**) was conducted by Martínez and coll. in 2018; the highest *M*_n_ of 155 kDa was achieved in C_2_H_4_Cl_2_ (0.1 mol% **Ru14**) [168].

### 3.3. Post-Modification of Unsaturated Polyesters and Application Prospects

#### 3.3.1. Post-Modification of Unsaturated Polyesters

**Figure 11:**
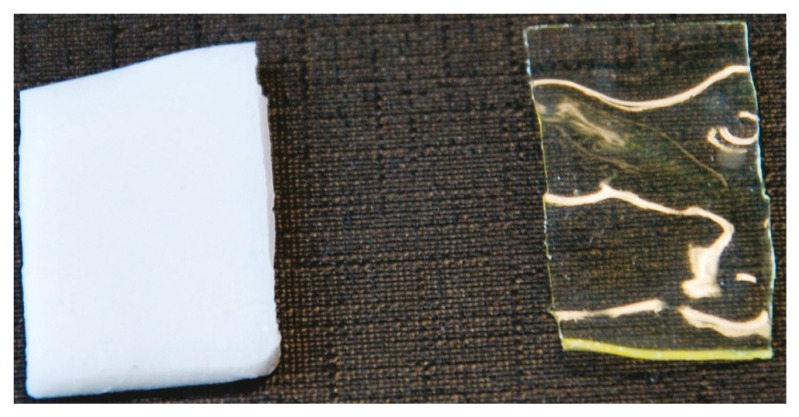
Poly(Gl-*co*-4MeCl) (75:25) before (**left**) and after (**right**) thermal cross-linking with dicumyl peroxide. Reprinted with permission from [131]. Copyright (2011) American Chemical Society.

A homopolymer of AL was subjected to thermal cross-linking with the use of dicumyl peroxide (DCP), which resulted in the complete disappearance of *T*_m_ and *T*_c_ peaks [129]. Poly(GL-*co*-MeCL) (75:25) had a *T*_m_ = 35.5 °C and represented opaque material; thermal cross-linking (DCP) resulted in the formation of transparent films (Figure 11). Poly(GL-*co*-DXO) had an enhanced biodegradability due to higher hydrophilicity [131].

The common methods of the post-functionalization of unsaturated polyesters are based on thiol-ene click chemistry. In 2011, poly(GL) was functionalized by the reaction with 6-mercapto-1-hexanol, butyl-3-mercapto propionate and N-acetylcysteamine, with the formation of polymers with a saturated backbone [169]. An attempt at pre-functionalization (by the AIBN-induced reaction of GL with N-acetylcysteamine) resulted in a cyclic substrate that was found to be inert in ROTEP [170]. However, the cyclic monomers obtained by the reaction of AL with *^n^*C_5_H_11_SH or HO(CH_2_)_6_SH, were active in a copolymerization with PDL, initiated by **Al1**, that resulted in functionalized PE-like copolymers [153]. Poly(GL-*co*-εCL) was cross-linked using EtC(CH_2_OC(O)CH_2_CH_2_SH)_3_, but in contrast with DCP cross-linking, more than 2 wt% of sol fraction was detected in copolymer containing 47 mol% of GL [132].

**Scheme 27:**
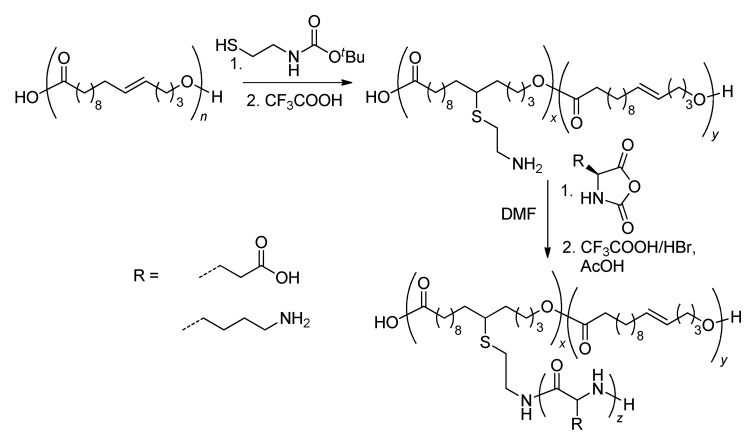
Synthesis of poly[(GL_20_-*g*-(AA)_z_] copolymers (AA—amino acid fragment). An 11-ene isomer of GL is depicted for simplicity [139,171].

An efficient method of cross-linking with the use of thiol-ene chemistry was proposed by Heise and coll. in 2017: the reactions of poly(GL) with HS(CH_2_)_5_SH or (CH_2_OC(O)CH_2_CH_2_SH)_2_ linkers were created using UV irradiation directly during the electrospinning (ES) [134]. Another non-standard approach was proposed by Mosnáček and coll.; transparent polymeric gels were obtained by the UV-initiated interaction of poly(GL) with a homopolymer of PEG-substituted SH-functionalized succinic acid [136]. The interaction of poly(GL) with *^t^*BuOC(O)NHCH_2_CH_2_SH resulted in a grafted polymer containing *N*-Boc substituents in side chains; after deprotection, derivatives of *L*-glutamic acid [139,171] and *L*-lysine [139] were obtained via condensation, with corresponding cyclic anhydride (Scheme 27). A similar synthetic strategy was used for the synthesis of poly(GL) containing oligo(*L*-alanine) fragments [172].

**Figure 12:**
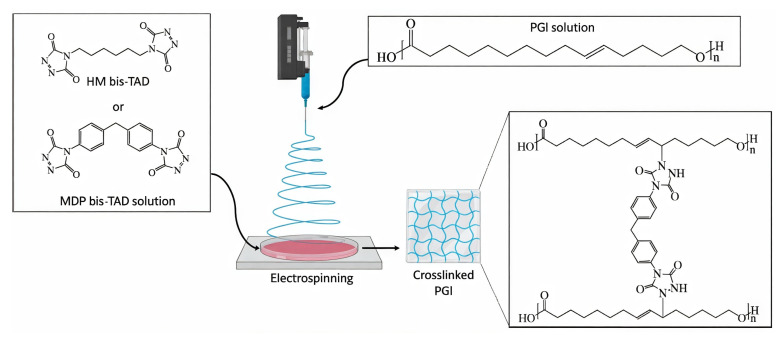
Electrospinning of poly(GL) into bis(triazolinedione) solutions. Reprinted with permission from [138]. Copyright (2019) MDPI AG.

Another approach to the functionalization of poly(GL) was based on the reaction with triazolinediones [138]. Cross-linking was performed during ES molding (Figure 12); non-crosslinked and crosslinked fibers had different filament and surface morphologies. To increase poly(GL) biocompatibility, the polymer was functionalized by HS(CH_2_)_6_OP(O)(OR)_2_ (R = Et, Ph); the ES of a 1:1 *w*/*w* mixture of functionalized poly(GL) and poly(PDL) resulted in the obtaining of scaffolds with an excellent morphology [173].

The thiol-ene reaction was used for the introduction of reactive –S(CH_2_)_6_OH fragments at the surface of poly(GL) films, with a subsequent reaction with a-bromoisobutyryl bromide [133]. Functionalized films were used as a substrate for the atom transfer radical polymerization (ATRP) of *^t^*Bu acrylate, followed by deprotection with the formation of polyacids. The surface modification of poly(GL-*co*-εCL) nanoparticles by *N*-acetylcysteine (model reactant) and bovine serum albumine (BSA) was described in [143]. Functionalization of poly(GL-*co*-εCL) by *N*-acetylcysteine in solution was carried out in [144,174] the degree of functionalization was 40–80 mol% depending on the GL/εCL ratio. Poly(GL-*co*-εCL) was also functionalized by a thiol-ene reaction with *L*-cysteine; copolymer was used for the preparation of non-toxic superparamagnetic iron oxide nanoparticles containing reactive –COOH groups [175]. Poly(GL-*co*-PDL) ES mats were used for *L*-cysteine grafting followed by chemical binding with gelatin via EDC/NHS chemistry to increase the hydrophilicity and biocompatibility of fibrous scaffolds [176].

Poly(HL) was transformed to corresponding polyoxirane (3-chlorobenzoperoxoic acid (mCPBA) in CH_2_Cl_2_) with subsequent cross-linking by a reaction with Na(CN)BH_3_/BF_3_∙Et_2_O that resulted in an increase in the *T*_m_ from 57.6 to 65.8 °C [154].

The exhaustive hydrogenation of copolymers obtained by the co-ROMP of AL and (*Z*)-cyclooctene (Scheme 26) resulted in the formation of PE-like materials with promising characteristics (see Section 3.2) [166].

#### 3.3.2. Cytotoxicity, Biocompatibility and Prospects of Biomedical Applications

A standard MTT test (3T3 mouse fibroblast cell line) revealed the absence of the cytotoxicity of AL and HL homopolymers [129]. ES-molded mats prepared from cross-linked poly(GL) showed a good biocompativility with mesenchymal stem cells (MSCs); the presence of S-containing linkers had no effect on cell viability [134].

In 2014, Heise and coll. synthesized poly(–COOH) functionalized films based on poly(GL) (see Section 3.3.2) and used these materials for the immobilization of proteins (fluorescent protein eGFP, Chitobiase enzyme) [133]. The obtained material demonstrated a high activity in the hydrolysis of N-acetylglucosamine.

BSA–poly(GL-*co*-εCL) nanoparticle conjugates were studied for cell uptake; a reduced internalization of the nanoparticles by Hela cells and macrophages was detected [143]. *N*-Acetylcysteine-functionalized poly(GL-*co*-εCL) demonstrated moderate antioxidant activity in both DPPH (EC_50_ = 4065 ± 157 μg∙mL^−1^) and ABTS (EC_50_ = 1553 ± 22 μg∙mL^−1^) assays (for *N*-acetylcysteine, the EC_50_ = 4.31 ± 0.03 μg∙mL^−1^ (DPPH) and EC_50_ = 137 ± 3 μg∙mL^−1^ (ABTS)) [144].

Poly(GL) was simultaneously functionalized by HS-containing antimicrobal dyes and cross-linked with (CH_2_OCH_2_CH_2_SH)_2_ via the thiol-ene reaction [140]. The resulting organogels showed excellent antimicrobial activity against *S. aureus* and *E. coli*.

The colloidal behavior of functionalized poly(GL)s containing ~20 GL fragments and ~5 fragments of *L*-glutamic acid or ~12 fragments of *L*-lysine was studied in [139]. In aqueous media, they formed pH-responsive micelles; derivatives of *L*-glutamic acid were studied as doxorubicin delivery articles, whereas *L*-lysine derivatives demonstrated an ability to form polyplexes with salmon testes DNA [139]. Experiments on gene delivery were not conducted during this study.

Based on poly(GL) (*M*_n_ = 51 kDa, *T*_m_ = 44 °C), bilayered films with regenerated cellulose nanofibers were prepared via layer-by-layer casting [177]. The bilayered films showed a keratinocyte growth far superior in comparison with pristine poly(globalide), demonstrating the high potential of these materials in skin tissue engineering.

**Figure 13:**
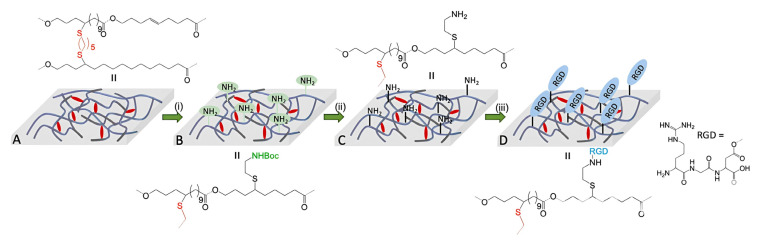
Reaction steps to produce RGD-functional electrospun fibers from crosslinked poly(globalide): (**A**) dithiol crosslinked PGL; (**B**) functionalization of crosslinked fibers with 2-(Boc-amino) ethanethiol (i); (**C**) Boc deprotection to produce amino functional fibers by TFA (ii); (**D**) coupling of RGD to amino functional fibers by EDC/NHS coupling (iii). Reprinted with permission from [178]. Copyright (2022) Wiley-VCH Verlag GmbH and Co.

Introducing RGD peptide to improve the cell-adhesive and proliferative characteristics of poly(GL) was performed by Heise and coll. [178]. ES mats were cross-linked by HS(CH_2_)_5_SH, functionalized by *^t^*BuOC(O)NH(CH_2_)_2_SH and, after deprotection, coupled with RGD peptide via 1-ethyl-3-(3-dimethylaminopropyl)carbodiimide and N-hydroxysuccinimide (EDC/NHS) chemistry (Figure 13). RGD-functionalized ES material showed a similar tensile strength as compared with non-functionalized cross-linked fibers but a higher elasticity. An in vitro test revealed the increased adhesion and proliferation of human mesenchymal stem cells.

Poly(LLA-*b*-GL-*b*-LLA) and poly(DLLA-*b*-GL-*b*-DLLA) with different ratios of lactate and GL fragments were used for the preparation of porous scaffolds for bone regeneration [150]. Chain-end functionalization using NHS, followed by binding with RGD peptide, increased the adhesion and proliferation of BM-MSCs and MC3T3-E1 cells.

#### 3.3.3. Other Possible Applications of Unsaturated (Co)Polyesters

**Figure 14:**
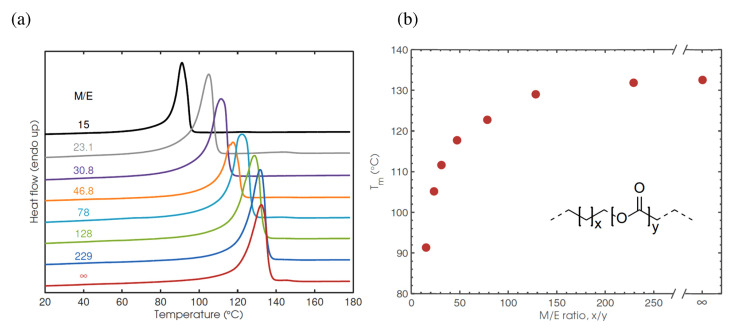
DSC thermograms of the saturated copolymers of AL and (*Z*)-cyclooctene (**a**) and a plot of the *T*_m_ as a function of M/E (**b**). Reprinted with permission from [166]. Copyright (2013) American Chemical Society.

Hydrogenated copolymers of AL and (*Z*)-cyclooctene with methylene-to-ester ratios (M/E) of 15–223 had a *T*_m_ ranging from 132.1 °C (hydrogenated (*Z*)-cyclooctene homopolymer) to 91.5 °C (M/E = 15) [166] (Figure 14). Hydrogenated HL homopolymer had a *T*_m_ = 47.6 °C [168]. Introducing ester groups into the PE backbone, besides reducing the *T*_m_ (and consequently molding temperature), may affect the biodegradability of the material. The results of these studies were used in further investigations of PE-like materials obtained from renewable feedstocks [179,180,181].

Copolymers of PDL with HO(CH_2_)_6_S-functionalized AL, containing both short- and long-chain branches, have demonstrated elastomeric behavior [153].

## 4. Conclusions

The RCM of alkenyl alkenoates, including derivatives of renewable raw materials and derivatives of undec-10-enoic and dec-9-enoic acids, represent an efficient method for the synthesis of UMs. Obvious progress has been made in the development of highly selective RCM catalysts that provide the formation of UMs with a given ring size and position and configuration of C=C bonds. Recent studies on the optimization of the synthesis of NHC Ru complexes (G-II, HG-II and their analogs) with the use of mechanochemical methods [81] improve the availability of the Ru catalysts of RCM and therefore RCM products. It is possible to expect that scalable approaches to newly discovered highly active and selective catalysts (CAAC, dithiolate derivatives and others) will also be developed in the near future.

The structural variety of UMs potentially entails the differences in their chemical properties. The ROTEP of Ums, with the formation of unsaturated polyesters that are capable of post-modification, appears a promising approach to new materials; however, numerous MUs with different structures are still unexplored. One possible reason is the unavailability of synthetic MUs in preparative amounts, but quite recently Grela and coll. Proposed an efficient method for the preparation of UMs, based on the shift of oligomer/UM equilibria by the separation of the reaction product by vacuum distillation [31,99,111]. Further improvement of this method requires the rational design of the catalysts and the optimization of the processes.

Solutions to these problems will allow the use of UMs as a starting compound in the synthesis of new materials with a variety of potential advanced applications, including the development of composite scaffolds, polymeric vehicles for drug and gene delivery and others.

## Figures and Tables

**Table 1:** Formation of 14-membered MLs via RCM of CH_2_=CH(CH_2_)_n_COO(CH_2_)_10−n_CH=CH_2_ (2 mM in CH_2_Cl_2_) catalyzed by **Ru4** (2 mol%) [52].

n	Time, h	Yield (Recovered Substrate), %	(*Z*)/(*E*) Ratio	Calc. (*Z*)/(*E*) Ratio ^1^
1	30	11 (41)	50:50	4:96
2	31	45 (13)	18:82	1:99
3	20	47 (26)	77:23	65:35
4	6	75 (5)	44:56	35:65
5	30	62 (11)	1:99	4:96
6	20	31 (28)	41:59	36:64
7	1.5	63 (9)	27:73	41:59
8	31	70 (5)	13:87	5:95
9	30	0 (66)	–	35:65

^1^ Calculated using MicroModel 4.5 [53].

**Table 2:** Experimental data on ring-closing metathesis of CH_2_=CH(CH_2_)_8_COO(CH_2_)_4_CH=CH_2_.

Catalyst	Substr. Conc., mM	Catalyst, mol%	Solvent	Time, h	Т, °С	Conv., %	Yield, %	(*Z*)/(*E*) Ratio	Ref.
**Ru2**	10	1	EtOAc	4	70	– ^1^	<5	20:80	[41]
**Ru3**	10	1	EtOAc	4	70	–	<5	20:80	[41]
**Ru4**	4.8	4	CH_2_Cl_2_	24	20	–	79	–	[50]
**Ru4**	2.7	1	supercritical CO_2_, d = 0.62 g∙mL^−1^	72	40	–	88	–	[56]
**Ru5**	5	0.05	C_6_H_6_	1; 4	40	12; 31	5; 21	–	[58]
**Ru6**	10	1	EtOAc	4	70	–	83	20:80	[41]
**Ru6**	5	0.003; 0.03	toluene	40 min	70	17; 99	12; 98	–; 24:76	[59]
**Ru7**	100; 5	5	CH_2_Cl_2_	0.25; 5	40	85; 99	10; 99	28:72	[49]
**Ru8**	10	1	EtOAc	4	70	–	79	20:80	[41]
**Ru9**	10	3	toluene (in air)	1.5	50	–	72	23:77	[60]
**Ru10**	8	0.0075	toluene	–	80	100	83	24:76	[61]
**Ru11**	--	2	CH_2_Cl_2_	19	40	–	72	24:76	[54]
**Ru12**	10	1	EtOAc	4	70	–	31	20:80	[41]
**Ru13**	5	5	CH_2_Cl_2_	1	22	–	–	–	[49]
**Ru14**	5	0.05	C_6_H_6_	1; 4	40	48	24; 33	–	[58]
**Ru14**	10	1	EtOAc	4	70	–	79	20:80	[41]
**Ru15**	10	1	EtOAc	4	70	–	42	20:80	[41]
**Ru15**	5	0.05	toluene; toluene 0.1% H_2_O	2	20	93; 43	87; 30	22:78	[62]
**Ru15**	5	1; 0.1	C_6_H_6_	1	20	100; 91	92; 80	22:78	[33]
**Ru15**	5	0.3	toluene with C_2_H_4_ rem.; toluene	2	70	77; 5	54; 4	26:74	[63]
**Ru15**	5	0.3	toluene	0.7	70	10	7	–	[59]
**Ru16**	5	0.3	toluene with C_2_H_4_ rem.; toluene	2	70	77; 8	69; 7	29:71	[63]
**Ru16**	5	0.3	toluene	20 min	70	5 ± 2	5 ± 2	–	[59]
**Ru16**	5	2.5	EtOAc	5	80	99	17	–	[67]
**Ru17**	5	0.05	toluene; toluene 0.1% H_2_O	2	20	92; 72	85; 62	28:72; 30:70	[62]
**Ru17**	5	0.3	toluene with C_2_H_4_ rem.; toluene	2	70	98; 87	91; 77	24:76; 73:27	[63]
**Ru18**	5	0.3	toluene with C_2_H_4_ rem.; toluene	2	70	90	85; 7	30:70	[63]
**Ru19**	10	1	EtOAc	4	70	–	43	20:80	[41]
**Ru20**	10	1	EtOAc	4	70	–	70	20:80	[41]
**Ru21**	10	1	EtOAc	4	70	–	76	20:80	[41]
**Ru23**	5	0.05	C_6_H_6_	1; 4	40	39; 56	29; 40	–	[58]
**Ru24**	8	0.0075	toluene with C_2_H_4_ rem.	–	80	100	88	25:75	[61]
**Ru24**	5	0.003	toluene	20 min	70	˂1	˂0.5	–	[59]
**Ru25**	8	0.005	toluene with C_2_H_4_ rem.	–	80	92	80	24:76	[61]
**Ru26**	10	1	EtOAc	29	70	–	<5		[41]
**Ru26**	10	1	EtOAc	4	100	–	8	20:80	[41]
**Ru26**	10	1	EtOAc; 20 mol% 1 M HCl	4	100	–	88/80 ^2^	20:80	[41]
**Ru26**	10	1	EtOAc; 10 mol% 1 M HCl	4	100	–	90/76	30:70	[41]
**Ru26**	10	0.1	EtOAc; 5 mol% 1 M HCl	4	100	–	88/76	20:80	[41]
**Ru26**	10	0.05	EtOAc; 2.5 mol% 1 M HCl	4	100	–	90/78	20:80	[41]
**Ru26**	20	0.05	EtOAc; 2.5 mol% 1 M HCl	4	100	–	76/67	20:80	[41]
**Ru26**	10	0.05	Me-THF; 2.5 mol% 1 M HCl	4	100	–	91/73	20:80	[41]
**Ru26**	10	0.05	(EtO)_2_CO; 2.5 mol% 1 M HCl	4	100	–	13/7	20:80	[41]
**Ru26**	10	0.05	iPrOH; 2.5 mol% 1 M HCl	4	100	–	26/20	20:80	[41]
**Ru27**	10	1	EtOAc; 2.5 mol% 1 M HCl	4	100	–	95/72	30:70	[41]
**Ru28**	10	1	EtOAc; 2.5 mol% 1 M HCl	1	100	–	93/73	40:60	[41]
**Ru29**	10	1	EtOAc; 2.5 mol% 1 M HCl	1	100	–	90/70	30:70	[41]
**Ru30**	10	1	EtOAc; 2.5 mol% 1 M HCl	4	100	–	93/73	30:70	[41]
**Ru31**	--	4	CH_2_C1_2_	55	40	–	81	24:76	[54]
**Ru32**	5	0.05	toluene 0; 0.01; 0.1 vol.% H_2_O	2	20	91; 49; 3	86; 36; 3	22:78–33:63	[62]
**Ru32**	5	1; 0.1	C_6_H_6_	1	20	100; 95	100; 85	22:78	[33]
**Ru32**	5	0.1	C_6_H_6_	0.25	60	99	66	24:76	[33]
**Ru32**	5	0.05	C_6_H_6_	2	23	75	72	–	[64]
**Ru35**	5	0.5	toluene 0; 0.1 vol.% H_2_O 0.01 bar	2	60	68; 31	64; 25	83:17; 85:15	[62]
**Ru37**	5	5	toluene 0; 0.1 vol.% H_2_O 0.01 bar	2	60	44; 52	17; 17	75:25; 70:30	[62]
**Ru43**	5	0.05	C_6_H_6_	1; 4	40	86; 94	80; 90	–	[58]
**Ru43**	5	0.003	toluene	20 min	70	10	10	–	[59]
**Ru43**	5	0.05	toluene	2	80	100	87	–	[68]
**Ru43**	20	0.05	toluene	2	80	100	68	–	[68]
**Ru44**	5	0.003	toluene	20 min	70	6	4	–	[59]
**Ru45**	5	0.0045	toluene	20 min	70	99	98	34:66	[59]
**Ru45**	10	0.0015	toluene	20 min	70	94	85	34:66	[59]
**Ru45**	20	0.002	toluene	20 min	70	94	62	36:64	[59]
**Ru45**	5	0.01; 0.0045	C_6_H_6_	2	40; 70	100; 99	94; 98	–	[64]
**Ru46**	5; 10	0.1; 0.05	toluene	6	80	99; 85	87; 81	30:70	[65]
**Ru46**	10; 25	0.01	toluene	6	80	55; 49	55; 45	30:70	[65]
**Ru46**	50; 100	0.01	toluene	6	80	50; 49	25; 25	30:70	[65]
**Ru47**	5	0.005	toluene	20 min	70	8	8	–	[59]
**Ru47**	5	0.025; 0.025	C_6_H_6_	2	70; 80	95; >99	91; 83	–	[64]
**Ru47**	5	0.025	toluene	1	75	95	91	35:65	[66]
**Ru48**	5	0.01; 0.002	C_6_H_6_	2	40	100; 56	92; 55	–	[64]

^1^ No data. ^2^ GC data or GC data/isolated yield.

**Table 3:** Experimental data on ring-closing metathesis of R^1^CH=CH(CH_2_)_n_COO(CH_2_)_m_CH=CHR^2^.

R^1^	n	m	R^2^	Cat.	Sub. Conc., mM	Cat., mol%	Solvent	Time, h	Т, °С	Yield, %	(*Z*)/(*E*) Ratio	Ref.
H	3	9	H	**Ru4**	4.8	4	CH_2_Cl_2_	23	20	62	– ^1^	[50]
H	2	8	H	**Ru4**	–	–	–	72	25	22	–	[32]
H	8	2	H	**Ru6**	3	–	CH_2_Cl_2_	40 min	40	>99	8:92	[55]
H	8	2	Et	**Ru6**	3	–	CH_2_Cl_2_	30 min	40	>99	9:91	[55]
H	3	9	H	**Ru6**	5	5	CH_2_Cl_2_	1	40	61	21:79	[45]
H	1	2	H	**Ru7**	5; 0.5	5	CH_2_Cl_2_	1	40	29; 78	0:100	[49]
H	4	2	H	**Ru7**	5; 0.5	5	CH_2_Cl_2_	0.5; 0.75	40	41; 95	41:59	[49]
H	8	2	H	**Ru7**	5	5	CH_2_Cl_2_	3	40	94	89:11	[49]
H	1	2	H	**Ru7**	5	5	CH_2_Cl_2_	30 min	40	~30	–	[73]
H	8	2	H	**Ru10**	40	0.005	toluene	–	80	30	9:91	[61]
H	2	9	H	**Ru10**	10	0.0075	toluene	–	80	50	10:90	[61]
H	0	10	H	**Ru14**	1	5	CH_2_Cl_2_	12	22	87	5:95	[78]
H	7	–(*R*)-CH(Me) (CH_2_)_2_CH=CH_2_		**Ru14**	–	–	toluene, C_6_F_6_	–	–	27	–	[79]
H	7	5	Et (*Z*-)	**Ru15**	1.5	2	toluene ^2^	5	50	87 ^3^	18:82	[42]
*n*-C_8_H_17_ (*Z*-)	7	5	Et (*Z*-)	**Ru15**	1.5	2	toluene ^2^	5	50	91 ^3^	22:78	[42]
*n*-C_8_H_17_ (*Z*-)	7	5	Et (*Z*-)	**Ru15**	1.5	2	EtOAc ^2^	5	77	91 ^3^	19:81	[42]
*n*-C_8_H_17_ (*Z*-)	7	5	Et (*Z*-)	**Ru15**	5	1	EtOAc ^2^	5	77	90 ^3^	19:81	[42]
*n*-C_8_H_17_ (*Z*-)	7	5	Et (*Z*-)	**Ru15**	10	1	EtOAc ^2^	24	77	81 ^3^	19:81	[42]
*n*-C_8_H_17_ (*Z*-)	7	5	Et (*Z*-)	**Ru15**	15	1	EtOAc ^2^	24	77	48 ^3^	20:80	[42]
*n*-C_8_H_17_ (*Z*-)	7	5	Et (*Z*-)	**Ru15**	21	0.5	EtOAc ^2^	24	77	43 ^3^	17:83	[42]
*n*-C_8_H_17_ (*Z*-)	7	5	Et (*Z*-)	**Ru15**	29	0.3	EtOAc ^2^	24	77	40 ^3^	17:83	[42]
*n*-C_8_H_17_ (*Z*-)	7	5	Et (*Z*-)	**Ru15**	40	0.1	EtOAc ^2^	24	77	35 ^3^	20:80	[42]
*n*-C_8_H_17_ (*Z*-)	7	5	Et (*Z*-)	**Ru15**	50	0.1	EtOAc ^2^	24	77	36 ^3^	17:83	[42]
*n*-C_8_H_17_ (*Z*-)	7	5	Et (*Z*-)	**Ru15**	100	0.1	EtOAc ^2^	24	77	16 ^3^	18:82	[42]
H	7	6	H	**Ru15**	1.5	2	toluene ^2^	5	50	77	31:69	[42]
H	7	–(CH_2_)_2_CHMe (CH_2_)_2_C=CMe_2_		**Ru15**	1.5	2	toluene ^2^	5	50	88	15:85	[42]
H	7	–(*Z*)-CH_2_C=C(Me) (CH_2_)_2_C=CMe_2_		**Ru15**	1.5	2	toluene ^2^	5	50	80	24:76	[42]
H	7	–(*E*)-CH_2_C=C(Me) (CH_2_)_2_C=CMe_2_		**Ru15**	1.5	2	toluene ^2^	5	50	87	13:87	[42]
H	7	–(*Z*)-(CH_2_)_5_ CH=CHEt		**Ru15**	1.5	2	toluene ^2^	5	50	87	18:82	[42]
H	7	4	H	**Ru15**	1.5	2	toluene ^2^	5	50	71	48:52	[42]
Oleyl (*n* = 7)		6	H	**Ru15**	1.5	2	toluene ^2^	5	50	90	31:69	[42]
Oleyl (*n* = 7)		–(CH_2_)_2_CHMe (CH_2_)_2_C=CMe_2_		**Ru15**	1.5	2	toluene ^2^	5	50	48	18:82	[42]
Oleyl (*n* = 7)		–(*Z*)-CH_2_C=C(Me) (CH_2_)_2_C=CMe_2_		**Ru15**	1.5	2	toluene ^2^	5	50	48	25:75	[42]
Oleyl (*n* = 7)		–(*E*)-CH_2_C=C(Me) (CH_2_)_2_C=CMe_2_		**Ru15**	1.5	2	toluene ^2^	5	50	40	16:84	[42]
Oleyl (*n* = 7)		–(*Z*)-(CH_2_)_5_ CH=CHEt		**Ru15**	1.5	2	toluene ^2^	5	50	91	22:78	[42]
Oleyl (*n* = 7)		4	H	**Ru15**	1.5	2	toluene ^2^	5	50	62	48:52	[42]
Me (*E*-)	6	6	H	**Ru15**	1.5	2	toluene ^2^	5	50	84	43:57	[43]
H	7	5	Me (*E*)-	**Ru15**	1.5	2	toluene ^2^	5	50	85	18:82	[43]
H	3	9	H	**Ru16**	5	0.5	AcOEt	70 min	70	99	25:75	[80]
Oleyl (*n* = 7)		5	Et (*Z*-)	**Ru17**	5	1	toluene	5	50	80	18:82	[81]
Me (*E*-)	6	7	Me (*E*-)	**Ru17**	1.5	2	toluene ^2^	5	50	84	–	[82]
Me (*E*-)	6	5	Et (*Z*-)	**Ru17**	1.5	2	toluene ^2^	5	50	84	–	[82]
Me (*E*-)	6	8	H	**Ru17**	1.5	2	toluene ^2^	5	50	86	–	[82]
H	7	5	Me (*E*-)	**Ru17**	1.5	2	toluene ^2^	5	50	85	–	[82]
H	8	2	H	**Ru24**	40	0.005	toluene	–	80	25	9:91	[61]
H	8	3	H	**Ru24**	8	0.01	toluene	–	80	12	–	[61]
H	2	9	H	**Ru24**	10	0.0075	toluene	–	80	56	9:91	[61]
H	8	2	H	**Ru25**	40	0.005	toluene	–	80	26	9:91	[61]
H	2	9	H	**Ru25**	10	0.0075	toluene	–	80	56	9:91	[61]
H	7	6	H	**Ru26**	10	1	EtOAc ^4^	4	77	70	–	[41]
H	8	6	H	**Ru26**	10	1	EtOAc ^4^	4	77	76	–	[41]
H	8	3	H	**Ru26**	10	1	EtOAc ^4^	4	77	39	–	[41]
H	8	9	H	**Ru31**	–	4	CH_2_Cl_2_	20	40	73	–	[54]
H	7	2	H	**Ru35**	3	7.5	C_2_H_4_Cl_2_	24	60	40	86:14	[48]
H	8	2	H	**Ru35**	3	7.5	C_2_H_4_Cl_2_	24	60	58	85:15	[48]
H	5	8	H	**Ru35**	3	7.5	C_2_H_4_Cl_2_	24	60	71	89:11	[48]
H	3	9	H	**Ru35**	3	7.5	C_2_H_4_Cl_2_	24	60	72	84:16	[48]
H	8	8	H	**Ru35**	3	7.5	C_2_H_4_Cl_2_	24	60	75	94:6	[48]
H	7	–(*R*)-CH(Me) (CH_2_)_2_CH=CH_2_		**Ru35**	–	–	toluene, C_6_F_6_	–	–	38	95:5	[79]
H	5	8	H	**Ru36**	3	7	C_2_H_4_Cl_2_	24	60	64	95:5	[47]
H	7	5	Et	**Ru37**	3	6	CH_2_Cl_2_	1	40	–	95:5	[83]
H	5	3	Me (*Z*-)	**Ru38**	3	6	CH_2_Cl_2_	1	40	70	>99:1	[84]
H	7	2	Et (*Z*-)	**Ru38**	3	6	CH_2_Cl_2_	1	40	98	95:5	[84]
H	8	2	Et (*Z*-)	**Ru38**	3	6	CH_2_Cl_2_	1	40	67	95:5	[84]
H	7	3	Me (*Z*-)	**Ru38**	3	6	CH_2_Cl_2_	1	40	72	98:2	[84]
H	8	3	Et (*Z*-)	**Ru38**	3	6	CH_2_Cl_2_	1	40	74	99:1	[84]
H	8	5	Et (*Z*-)	**Ru38**	3	6	CH_2_Cl_2_	1	40	75	95:5	[84]
Me (*E*-)	8	4	Me (*E*-)	**Ru39**	5	7.5	THF	24	35	66 ^3^	1:99	[85]
Me (*E*-)	5	3	Me (*E*-)	**Ru40**	5	7.5	THF	5	35	60 ^3^	1:99	[85]
Me (*E*-)	7	2	Me (*E*-)	**Ru40**	5	7.5	THF	5	35	75 ^3^	1:99	[85]
Me (*E*-)	8	2	Me (*E*-)	**Ru40**	5	7.5	THF	5	35	65 ^3^	1:99	[85]
Me (*E*-)	8	3	Me (*E*-)	**Ru40**	5	7.5	THF	5	35	70 ^3^	1:99	[85]
Me (*E*-)	7	3	Me (*E*-)	**Ru40**	5	7.5	THF	5	35	67 ^3^	1:99	[85]
Me (*E*-)	8	4	Me (*E*-)	**Ru40**	5	7.5	THF	5	35	70 ^3^	1:99	[85]
Me (*E*-)	8	6	Me (*E*-)	**Ru40**	5	7.5	THF	5	35	63 ^3^	1:99	[85]
H	3	9	H	**Ru40**	100/5 ^5^	4	THF	12	35	56	96:4	[86]
H	6	4	H	**Ru40**	100/5 ^5^	4	THF	12	35	67	98:2	[86]
H	4	7	H	**Ru40**	100/5 ^5^	4	THF	12	35	86	98:2	[86]
H	4	8	H	**Ru40**	100/5 ^5^	4	THF	12	35	70	98:2	[86]
H	6	9	H	**Ru40**	100/5 ^5^	4	THF	12	35	65	98:2	[86]
H	8	9	H	**Ru40**	100/5 ^5^	4	THF	12	35	60	96:4	[86]
H	7	5	Et (*Z*-)	**Ru41**	3	6	CH_2_Cl_2_	1	40	<5	–	[83]
H	7	8	Me	**Ru42**	20	2	toluene	1	100	83	9:91	[72]
H	2	7	Me	**Ru42**	20	2	toluene	1	100	32	24:76	[72]
H	2	8	Me	**Ru42**	20	2	toluene	1	100	40	10:90	[72]
H	3	8	Me	**Ru42**	20	2	toluene	1	100	48	34:66	[72]
H	4	8	Me	**Ru42**	20	2	toluene	1	100	52	14:86	[72]
H	7	5	Me	**Ru42**	20	2	toluene	1	100	55	24:76	[72]
H	3	10	Me	**Ru42**	20	2	toluene	1	100	71	14:86	[72]
H	4	10	Me	**Ru42**	20	2	toluene	1	100	68	17:83	[72]
H	7	7	Me	**Ru42**	20	2	toluene	1	100	75	15:85	[72]
H	4	11	Me	**Ru42**	20	2	toluene	1	100	81	6:94	[72]
H	5	11	Me	**Ru42**	20	2	toluene	1	100	78	28:72	[72]
H	7	10	Me	**Ru42**	20	2	toluene	1	100	62	19:81	[72]
H	7	11	Me	**Ru42**	20	2	toluene	1	100	59	27:73	[72]

^1^ No data. ^2^ 4 mol% TFQ. ^3^ Isolated yield. ^4^ 2.5 mol% HCl. ^5^ 20 equiv. of (*Z*)-but-2-ene was added at the first stage; after elimination of the excess of (*Z*)-but-2-ene, the mixture was diluted to 5 mM.

**Table 4:** Synthesis of UMs via ring-closing depolymerization of products of ADMET polymerization of CH_3_(CH_2_)_7_CH=CH(CH_2_)_7_COO(CH_2_)_n_CH=CHR (200 mM, 110 °C, 8 h) [31].

n	R	Cat	Cat. mol%	Diluent	Yield, %	Sel., %	(*Z*)/(*E*) Ratio
5	Et	**Ru53**	0.5	paraffin oil	97	94	24/66
5	Et	**Ru53**	0.5	paraffin wax	91	86	24/66
5	Et	**Ru53**	0.5	polyethylene	26	92	22/78
5	Et	**Ru53**	0.5	ionic liquid ^1^	33	77	23/77
5	Et	**Ru53**	0.5	PAO4 ^2^	97	96	23/77
5	Et	**Ru53**	0.5	PAO6 ^3^	99	95	23/77
2	H	**Ru52**	2	PAO6	73	96	22/78
–CH_2_CHMe(CH_2_)_2_C=CMe_2_		**Ru53**	2	paraffin	56	92	19/81
5	H	**Ru53**	0.5	paraffin	60	93	24/66
6	H	**Ru52**	1	PAO6	86	94	32/68
8	*n*-C_8_H_17_	**Ru53**	2	paraffin	55	81	23/77

^1^ 1-butyl-2,3-dimethylimidazolium hexafluorophosphate. ^2^ Hydrogenated dec-9-ene trimer. ^3^ Hydrogenated dec-9-ene tetramer.

**Table 5:** RCM of CH_2_=CH(CH_2_)_8_COO(CH_2_)_n_CH=CH_2_ (5 mM in CH_2_Cl_2_, 40 °C, 5 mol% of the catalysts) [100].

n	Ring Size	Catalyst	Time, h	Yield, %	(*Z*)/(*E*) Ratio
2	14	**Ru14**	6	74	9:91
		**Ru56a**		83	7:93
		**Ru56b**		79	8:92
		**Ru56c**		61	8:92
4	16	**Ru14**	5	53	22:78
		**Ru56a**		96	23:77
		**Ru56b**		89	23:77
		**Ru56c**		77	22.78:
8	20	**Ru14**	6	29	32:68
		**Ru56a**		62	30:70
		**Ru56b**		55	29:71
		**Ru56c**		28	28:72
9	21	**Ru14**	8	19	31:69
		**Ru56a**		68	30:70
		**Ru56b**		62	30:70
		**Ru56c**		28	30:70

**Table 6:** RCM of CH_2_=CH(CH_2_)_3_COO(CH_2_)_9_CH=CH_2_ catalyzed by **Ru6** and Croup 6 metal catalysts (5 mM in toluene, 22 °C) [45].

Catalyst	Cat. Loading, mol%	Pressure, Torr	Conv., %	Yield, %	(*Z*)/(*E*) Ratio
**Ru6** ^1^	5	760	75	61	21:79
**Mo1**	5	760	85	60	22:78
**Mo1**	5	7	96	58	21:79
**Mo4**	5	760	56	45	70:30
**Mo4**	5	7	97	56	77:23
**Mo5**	5	7	91	55	72:28
**Mo6**	3	7	80	62	85:15
**Mo6**	1.2	7	75	56	92:8
**W1**	5	7	80	62	91:9
**W4**	5	7	14	10	95:5

^1^ The reaction was carried out in CH_2_Cl_2_ at 40 °C.

**Table 7:** Enzyme-catalyzed ROTEP and copolymerization of UMs. For comonomer abbreviations, see Scheme 21 and Scheme 22.

UM	Comon. (C)	[UM]/[C] Ratio ^1,2^	Catalyst (Loading, mg∙g^−1^)	Reaction Medium	Time, h	T, °C	Yield, %	*M*_n_, kDa	Ð_M_	Ref.
GL	–	–	Novozym 435 ^3^ (21)	toluene	4	60	~70	24.2	1.92	[129]
GL	–	–	Novozym 435 (40)	toluene	24	60	80–90	24	1.9	[130,131]
GL	–	–	Novozym 435 (40)	toluene	24	60	~70	15	2.8	[132]
GL	–	–	Novozym 435 (210)	toluene	4	60	~60	16	2.5	[133]
GL	–	–	Novozym 435 (40)	toluene	4	60	~60	25	–	[134]
GL	–	–	free *Candida antarctica* B in sorbitol	C_16_H_34_/H_2_O/em. ^4^	5	60	–	3.6	2.1	[135]
GL	–	–	Novozym 435	bulk	–	–	–	6.35	3.4	[136]
GL	–	–	NS 88011 (20)	toluene	2	60	–	20.9	4.7	[137]
GL	–	–	NS 88011 (100)	toluene	2	60	–	31.7	3.8	[137]
GL	–	–	Novozym 435 (60)	toluene	4	60	80	20	3.5	[138]
GL	–	–	Novozym 435 (210)	toluene	4	60	97	4.7	–	[139]
GL	–	–	*Candida antarctica* B on Immobead 150 ^5^	toluene	4	60	69	30.7	1.38	[140]
GL	–	–	Novozym 435 (60)	sc CO_2_^6^	2	65	85	15.2	1.66	[141]
GL	–	–	Novozym 435 (60)	sc propane ^6^	2	65	72	16.6	2.41	[141]
AL	–	–	Novozym 435 (21)	toluene	4	60	~70	18.5	1.94	[129]
AL	–	–	Novozym 435 (40)	toluene	24	60	80–90	24	1.9	[130,131]
GL	DXO	9–0.4 wt ^1^	Novozym 435 (40)	toluene	24	60	80–90	11–44	1.8–2.5	[130,131]
GL	MeCL	9–0.4 wt ^1^	Novozym 435 (40)	toluene	24	60	80–90	6–18	2.0–2.5	[130,131]
GL	εCL	0.89–0.11 ^2^	Novozym 435 (40)	toluene	24	60	~70	15–23	2.4–3.3	[132]
GL	εCL	0.05–4.3 ^1^	Novozym 435 (50)	sc CO_2_^6^	–	65	–	up to 25	–	[142]
GL	εCL	1 ^1^	Novozym 435 (50)	sc CO_2_	2	65	–	–	–	[143]
GL	εCL	0.1–9 wt ^1^	Novozym 435 (50)	sc CO_2_	2	65	–	–	–	[144]
AL	CE4O	3–0.4 wt ^1^	Novozym 435 (40)	toluene	24	60	80–90	8–14	1.4–2.0	[130]
AL	PDL	1:1 ^1,2^	Novozym 435 (100)	toluene	24	70	–	8.2	–	[145]

^1^ In feed. ^2^ In copolymer. ^3^ Novozym 435—*Candida antarctica* Lipase B immobilized on cross-linked polyacrylate beads. ^4^ Lutensol AT50 emulsifier was used. ^5^ Immobead 150—epoxy-containing methacrylate polymer. ^6^ Supercritical CO_2_.

**Table 8:** Synthesis of UM (co)polymers using non-enzymatic catalysis.

UM	Comon. (C)	[UM]/[C] Ratio ^1,2^	Catalyst (Loading)	Reaction Medium	Time, h	T, °C	Yield, %	*M*_n_^SEC^, kDa	Ð_M_	Ref.
GL	–	–	DBSA ^3^ (2 mol%)	in bulk	72	80	>98	7.8	2.55	[151]
GL	–	–	CF_3_SO_3_H (10 mol%)	in bulk	7	80	>98	n.d. ^4^	n.d.	[151]
GL	–	–	DBSA (10 mol%)	H_2_O/C_16_H_34_/em. ^5^	24	80	88	1.4	–	[151]
AL	–	–	DBSA (10 mol%)	in bulk	24	80	>98	6.3	2.94	[151]
AL	–	–	CF_3_SO_3_H (10 mol%)	in bulk	7	80	>98	n.d.	n.d.	[151]
AL	–	–	DBSA (10 mol%)	H_2_O/C_16_H_34_/em.	24	80	85	1.66	–	[151]
AL	–	–	**Al1** (1 mol%)	1 M in toluene	140	100	>98	12.1	6.7	[152]
AL	–	–	**Al1** (0.2 mol%)	in bulk	16	100	–	49	2.7	[153]
HL	–	–	**Al2** (1 mol%)	xylenes	27	100	60	15.4 ^6^	1.6	[154]
HL	–	–	**Al2** (0.4 mol%)	xylenes	27	130	60	36.3 ^6^	1.6	[154]
HL	–	–	**Al2** (0.4 mol%)	xylenes	27	130	45	12.6 ^6^	1.8	[155]
HL	–	–	**Zn1** (0.5 mol%)	toluene	24	100	70	12.6 ^6^	1.7	[156]
RL	–	–	Me_3_SiONa (2 mol%)	THF	114	40	–	1.4	1.1	[157]
HL	εCL	1	**Al2** (1 mol%)	xylenes	29	100	75	15.8 ^6^	1.6	[154]
HL	εCL	0.33	**Al2** (1 mol%)	xylenes	29	100	60	39.2	1.8	[155]
HL	εCL	0.1–1	**Zn1** (0.5 mol%)	toluene	24	100	50–70	14.7–18.2	1.5–1.6	[156]
HL	PDL	0.1–1	**Zn1** (0.5 mol%)	toluene	24	100	42–63	12.3–21.4	1.5–1.6	[156]
AL	PDL	0.05–1	**Al1** (0.2 mol%)	in bulk	16	100	–	48–87	2.2–2.6	[153]
RL	LLA	0.07–0.2	Sn(Oct)_2_ (1.5 wt%)	toluene	4	135	–	4.7–9.9	1.3–1.5	[157]

^1^ In reaction mixture. ^2^ In copolymer. ^3^ Dodecylbenzene sulfonic acid. ^4^ Not determined, high-MW polymer. ^5^ A3065 emulsifier was used. ^6^ Determined by ^1^H NMR.

## Abbreviations

The following abbreviations are used in this manuscript:

ADMET	Acyclic diene metathesis
AIBN	α,α′-Azoisobutyronitrile, 2,2′-azobis(2-methylpropionitrile)
AL	Ambrettolide (oxacycloheptadec-10-en-2-one)
BPO	Benzoyl peroxide
CAAC	Cyclic alkyl amino carbene
εCL	ε-Caprolactone
DCP	Dicumyl peroxide
DOSY	Diffusion ordered spectroscopy
EDC	1-ethyl-3-(3-dimethylaminopropyl)carbodiimide
ES	Electrospinning
FAMEs	Fatty acid methyl esters
G-I	First-generation Grubbs catalyst
G-II	Second-generation Grubbs catalyst
GC	Gas chromatography
GL	Globalide (oxacyclohexadec-12-en-2-one)
HG-II	Second-generation Hoveyda–Grubbs catalyst
HL	ω-6-Hexadecenlactone (oxacycloheptadec-7-en-2-one)
LA	Lactide
LLA	*L*-lactide
mCPBA	3-Chlorobenzoperoxoic acid
MO	Methyl oleate
MS	Mass spectrometry
NHC	N-heterocyclic carbene
NHS	*N*-hydroxysuccinimide
PDL	ω-Pentadecalactone (oxacyclohexadecan-2-one)
PE	Polyethylene
RA	Ricinoleic acid
RCM	Ring-closing metathesis
RL	Ricinoleic lactone ((*R*,*Z*)-13-hexyloxacyclotridec-10-en-2-one)
ROMP	Ring-opening metathesis polymerization
ROTEP	Ring-opening transesterification polymerization
Sn(Oct)_2_	Tin(II) 2-ethylhexanoate
TFQ	2,3,5,6-Tetrafluorocyclohexa-2,5-diene-1,4-dione
TMC	1,3-Dioxan-2-one (trimethylene carbonate)
TON	Turnover number, the molar ratio of the converted reactant and the catalyst
*T*_c_	Crystallization temperature
*T*_m_	Melting temperature
UM	Unsaturated macrolactone
